# Novel Pyrazole-Hydrazone Derivatives Containing an Isoxazole Moiety: Design, Synthesis, and Antiviral Activity

**DOI:** 10.3390/molecules23071798

**Published:** 2018-07-20

**Authors:** Zaibo Yang, Pei Li, Xiuhai Gan

**Affiliations:** 1 School of Chemistry and Chemical Engineering, Qiannan Normal University for Nationalities, Guizhou, Duyun 558000, China; 2 Qiandongnan Engineering and Technology Research Center for Comprehensive Utilization of National Medicine, Kaili University, Guizhou, Kaili 556011, China; pl19890627@126.com; 3 College of Chemistry and Life Science, Guizhou Normal College, Guizhou, Guiyang 550018, China; gxh200719@163.com

**Keywords:** pyrazole-hydrazone, isoxazole, antiviral activity, tobacco mosaic virus

## Abstract

In this study, a series of novel pyrazole-hydrazone derivatives containing an isoxazole moiety were synthesized. Antiviral bioassays indicated that some of the title compounds exhibited better in vivo antiviral activities against tobacco mosaic virus (TMV). In particular, compounds **6a**, **6c** and **6q** exhibited the best curative activity, protection activity, and inactivation activity against TMV, respectively, which were superior to those of Ningnanmycin. This study demonstrated that this series of novel pyrazole-hydrazone derivatives containing an isoxazole amide moiety could effectively control TMV.

## 1. Introduction

Plant viruses cause diseases in a wide range of crop plant species and annually contribute to an estimated $600 billion in economic loss worldwide [1]. In recent years, progress has been made in screening for high-activity antiviral agents in natural products in China. For example, Ningnanmycin, isolated from *Strepcomcesnoursei* var. *Xichangensis*, was found to be more effective than existing products in the treatment of plant viruses such as tobacco mosaic virus (TMV) and cucumber mosaic virus (CMV) [2]. Meanwhile, Ribavirin, a successful antiviral agent, is widely used to prevent TMV disease. However, the use of Ningnanmycin and Ribavirin in field trials is unsuitable because of its unsatisfactory curative rates and high control costs [3]. Therefore, identification of new effective antiviral agents remains a significant challenge [4]. 

In recent years, literature revealed that pyrazole derivatives had a wide range of biological activities, such as antibacterial [5,6,7], fungicidal [8,9,10], insecticidal [10,11,12,13], antiviral [14,15], and herbicidal [16,17] activities. In our previous work, we reported a series of pyrazole derivatives which had better insecticidal activity against *Plutella xylostella* [18]. Meanwhile, hydrazone, a highly efficient pharmacophore, has attracted more and more attention in the pesticides areas due to its broad-activities, such as antibacterial [19], fungicidal [20,21], insecticidal [20,22,23], and antiviral [20,24] activities. A few of the pyrazole-hydrazone derivatives have also been reported and possess various biological activities such as antibacterial [25], antifungal [21], anticancer [26], antioxidant [27], anti-inflammatory [28], antitumor and antiangiogenesis [29] and antiplatelet activities [30]. In addition, isoxazole, a five membered heterocyclic ring, was an important scaffold for synthesis of various natural compounds and their congeners and had a broad spectrum of pharmacological activities like antibacterial [31], antifungal [9,32,33], insecticide [34,35], and antiviral [36] activities. 

Motivated by the above-mentioned findings and in continuation of our investigation, to discover new potentially active agents, in this study, a series of novel pyrazole-hydrazone derivatives containing an isoxazole moiety were designed and synthesized using a 3-substituted phenyl-5-methyl isoxazole-4-carboxylic acid as the starting material. The antiviral bioassay results indicated that most of the title compounds exhibited excellent in vivo antiviral activities against TMV. To the best of our knowledge, it is the first report on pyrazole-hydrazone derivatives containing an isoxazole moiety with potent antiviral activity against TMV.

## 2. Results and Discussion

### 2.1. Chemistry

**Scheme 1:**
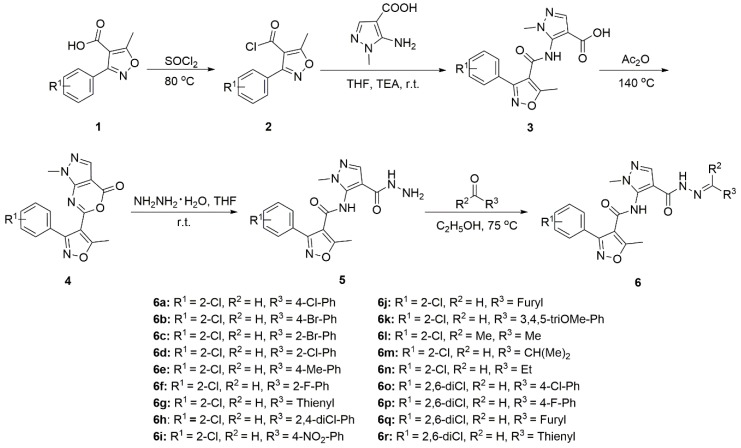
Synthetic route of the title compounds **6a**–**6r**.

Using 3-(2-chlorophenyl)-5-methyl isoxazole-4-carboxylic acid and 3-(2,6-dichlorophenyl)-5-methyl isoxazole-4-carboxylic acid as the starting materials, as shown in Scheme 1, the target compounds **6a**–**6r** were obtained in five steps with yields of 85.9–91.2%. The physical characteristics, IR, ^1^H-NMR, ^13^C-NMR, and elemental analysis data for all the synthesized compounds are shown below. The ^1^H-NMR and ^13^C-NMR for all the synthesized compounds are shown in Supplementary Materials.

*Data for N-(4-(2-(4-chlorobenzylidene)hydrazinecarbonyl)-1-methyl-1H-pyrazol-5-yl)-3-(2-chlorophenyl)-5-methylisoxazole-4-carboxamide* (**6a**). White solid; m.p. 141–143 °C; yield 87.9%; ^1^H-NMR (500 MHz, Dimethylsulfoxide-*d*_6_ (DMSO-*d*_6_), ppm) *δ*: 11.54 (s, 1H, isoxazole–CONH–), 10.32 (s, 1H, pyrazole–CONH–), 8.31 (s, 1H, –N=CH–), 8.01 (s, 1H, pyrazole-H), 7.74–7.47 (m, 8H, Ar–H), 3.65 (s, 3H, pyrazole–CH_3_), 2.85 (s, 3H, –CH_3_). ^13^C-NMR (125 MHz, DMSO-*d*_6_, ppm) *δ*: 171.53, 160.66, 158.43, 147.02, 145.78, 144.08, 140.42, 140.78, 139.24, 137.88, 137.67, 132.84, 132.15, 130.25, 130.13, 129.97, 127.94, 127.50, 127.26, 113.72, 109.57, 36.66, 12.99. IR (KBr, cm^−1^) *ν*: 3404.36, 3282.84, 2933.73, 1716.65, 1699.29, 1683.86, 1653.00, 1635.64, 1627.92, 1595.13, 1575.84, 1558.48, 1541.12, 1521.84, 1506.41, 1489.08, 1473.62, 1458.18, 1436.97, 1411.89, 1375.25, 1303.88, 1253.75, 1219.01. Anal. Calc. for C_23_H_18_Cl_2_N_6_O_3_: C, 55.55%; H, 3.65%; N, 16.90%; Found: C, 55.81%; H, 3.79%; N, 17.08%.

*Data for 3-(2-chlorophenyl)-N-(4-(2-(4-bromobenzylidene)hydrazinecarbonyl)-1-methyl-1H-pyrazol-5-yl)-5-methylisoxazole-4-carboxamide* (**6b**). White solid; m.p. 236–237 °C; yield 90.1%; ^1^H-NMR (500 MHz, DMSO-*d*_6_, ppm) *δ*: 11.57 (s, 1H, isoxazole–CONH–), 10.35 (s, 1H, pyrazole–CONH–), 8.29 (s, 1H, –N=CH–), 8.01 (s, 1H, pyrazole-H), 7.85–7.85 (d, 1H, *J* = 3.45 Hz, Ar–H), 7.66–7.45 (m, 7H, Ar–H), 3.66 (s, 3H, pyrazole–CH_3_), 2.86 (s, 3H, –CH_3_). ^13^C-NMR (125 MHz, DMSO-*d*_6_, ppm) *δ*: 171.46, 160.67, 158.38, 145.62, 137.80, 134.21, 132.87, 132.38, 132.36, 132.17, 132.16, 131.80, 130.14, 129.37, 129.19, 127.96, 127.93, 123.57, 113.57, 109.52, 36.55, 12.99. IR (KBr, cm^−1^) *ν*: 3444.87, 326.91, 2933.73, 1716.65, 1699.29, 1683.86, 1662.64, 1653.00, 1635.64, 1608.63, 1575.84, 1558.48, 1541.12, 1521.84, 1506.41, 1489.08, 1473.62, 1458.18, 1436.97, 1429.25, 1398.39, 1319.31, 1301.95. Anal. Calc. for C_23_H_18_ClBrN_6_O_3_: C, 50.99%; H, 3.35%; N, 15.51%; Found: C, 51.09%; H, 3.39%; N, 15.55%.

*Data for 3-(2-chlorophenyl)-N-(4-(2-(2-bromobenzylidene)hydrazinecarbonyl)-1-methyl-1H-pyrazol-5-yl)-5-methylisoxazole-4-carboxamide* (**6c**). White solid; m.p. 181–183 °C; yield 89.2%; ^1^H-NMR (500 MHz, DMSO-*d*_6_, ppm) *δ*: 11.69 (s, 1H, isoxazole–CONH–), 10.30 (s, 1H, pyrazole–CONH–), 8.61 (s, 1H, –N=CH–), 7.97 (s, 1H, pyrazole–H), 7.91 (d, 1H, *J* = 7.45 Hz, Ar–H), 7.63 (d,1H, *J* = 8.00 Hz, Ar–H), 7.54–7.38 (m, 5H, A–H), 7.31 (t, 1H, *J* = 14.85 Hz, Ar–H), 3.59 (s, 3H, pyrazole–CH_3_), 2.79 (s, 3H, –CH_3_). ^13^C-NMR (125 MHz, DMSO-*d*_6_, ppm) *δ*: 160.56, 158.40, 158.18, 152.48, 147.87, 144.98, 137.96, 137.71, 133.63, 132.75, 132.10, 131.86, 130.07, 128.58, 127.88, 127.71, 127.63, 123.88, 113.69, 113.58, 112.30, 36.61, 12.91. IR (KBr, cm^−1^) *ν*: 3446.79, 3298.28, 2933.73, 1716.65, 1699.29, 1683.86, 1670.35, 1653.00, 1635.64, 1575.84, 1558.48, 1541.12, 1521.84, 1506.41, 1489.08, 1473.62, 1458.18, 1436.97, 1411.89, 1398.39, 1361.74, 1338.60, 1319.31, 1298.09, 1253.73, 1220.94. Anal. Calc. for C_23_H_18_ClBrN_6_O_3_: C, 50.99%; H, 3.35%; N, 15.51%; Found: C, 51.06%; H, 3.41%; N, 15.57%.

*Data for 3-(2-chlorophenyl)-N-(4-(2-(2-chlorobenzylidene)hydrazinecarbonyl)-1-methyl-1H-pyrazol-5-yl)-5-methylisoxazole-4-carboxamide* (**6d**). White solid; m.p. 170–171 °C; yield 88.7%; ^1^H-NMR (500 MHz, DMSO-*d*_6_, ppm) *δ*: 11.64 (s, 1H, isoxazole–CONH–), 10.28 (s, 1H, pyrazole–CONH–), 8.66 (s, 1H, N=CH–), 7.97 (s, 1H, pyrazole–H), 7.53–7.37 (m, 8H, Ar–H), 3.59 (s, 3H, pyrazole–CH_3_), 2.79 (s, 3H, –CH_3_). ^13^C-NMR (125 MHz, DMSO-*d*_6_, ppm) *δ*: 171.52, 160.65, 158.51, 142.75, 140.37, 138.09, 137.70, 133.55, 132.82, 132.16, 131.88, 131.73, 130.46, 130.14, 128.17, 127.95, 127.84, 127.33, 127.14, 113.71, 109.36, 36.66, 12.97. IR (KBr, cm^−1^) *ν*: 3444.87, 3286.70, 2933.73, 1716.65, 1695.43, 1683.86, 1670.35, 1653.00, 1635.64, 1575.84, 1558.48, 1541.12, 1521.84, 1506.41, 1498.69, 1473.62, 1458.18, 1436.97, 1419.61, 1398.39, 1361.74, 1340.53, 1319.31, 1251.80, 1219.01. Anal. Calc. for C_23_H_18_Cl_2_N_6_O_3_: C, 55.55%; H, 3.65%; N, 16.90%; Found: C, 55.70%; H, 3.79%; N, 17.24%.

*Data for 3-(2-chlorophenyl)-5-methyl-N-(1-methyl-4-(2-(4-methylbenzylidene)hydrazinecarbonyl)-1H-pyrazol-5-yl)isoxazole-4-carboxamide* (**6e**). White solid; m.p. 221–223 °C; yield 89.8%; ^1^H-NMR (500 MHz, DMSO-*d*_6_, ppm) *δ*: 11.38 (s, 1H, isoxazole–CONH–), 10.27 (s, 1H, pyrazol–CONH–), 8.24 (s, 1H, –N=CH–), 7.97 (s, 1H, pyrazole–H), 7.55–7.42 (m, 6H, Ar–H), 7.22 (s, 1H, Ar–H), 7.20 (s, 1H, Ar–H), 3.62 (s, 3H, pyrazole–CH_3_), 2.81(s, 3H, –CH_3_), 2.28 (s, 3H, Ar–CH_3_). ^13^C-NMR (125 MHz, DMSO-*d*_6_, ppm) *δ*: 193.16, 171.80, 171.53, 163.39, 160.66, 158.43, 147.02, 145.78, 144.08, 140.42, 140.28, 140.08, 139.24, 137.88, 137.67, 134.52, 132.84, 132.15, 130.25, 130.13, 129.97, 129.65, 127.94, 127.50, 127.26, 113.72, 109.57, 108.03, 36.66, 21.55, 12.99. IR (KBr, cm^−1^) *ν*: 3444.87, 3203.76, 3030.17, 2933.73, 1699.29, 1683.86, 1662.64, 1653.00, 1635.64, 1602.85, 1577.77, 1558.48, 1541.12, 1521.84, 1506.41, 1473.62, 1456.26, 1436.97, 1409.96, 1398.39, 1375.25, 1301.95, 1246.02, 1219.01. Anal. Calc. for C_24_H_21_ClN_6_O_3_: C, 60.44%; H, 4.44%; N, 17.62%; Found: C, 60.53%; H, 4.48%; N, 17.73%.

*Data for 3-(2-chlorophenyl)-N-(4-(2-(2-fluorobenzylidene)hydrazinecarbonyl)-1-methyl-1H-pyrazol-5-yl)-5-methylisoxazole-4-carboxamide* (**6f**). White solid; m.p. 180–182 °C; yield 88.3%; ^1^H-NMR (500 MHz, DMSO-*d*_6_, ppm) *δ*: 11.62 (s, 1H, isoxazole–CONH–), 10.36 (s, 1H, pyrazole–CONH–), 8.57 (s, 1H, –N=CH–), 8.02 (s, 1H, pyrazole–H), 7.93–7.90 (t, 1H, *J* = 16.60 Hz, Ar–H), 7.60–7.28(m, 7H, Ar–H), 3.66 (s, 3H, pyrazole–CH_3_), 2.85 (s, 3H, –CH_3_). ^13^C-NMR (125 MHz, DMSO-*d*_6_, ppm) *δ*: 171.25, 160.15 (d, 1C, 2-F-Ph-C), 158.14, 139.27, 137.76, 137.41, 132.54, 132.04, 131.88, 129.86, 127.65, 127.54, 126.77, 126.49, 125.21, 122.11, 116.33, 116.18, 113.43, 109.10, 36.42, 12.69. IR (KBr, cm^−1^) *ν*: 3444.87, 3167.12, 2933.73, 1699.29, 1683.86, 1653.00, 1635.64, 1616.35, 1602.85, 1575.84, 1558.48, 1541.12, 1521.84, 1506.41, 1489.08, 1473.62, 1436.97, 1419.61, 1398.39, 1379.10, 1363.67, 1319.31, 1242.16, 1215.15. Anal. Calc. for C_23_H_18_ClFN_6_O_3_: C, 57.45%; H, 3.77%; N, 17.48%; Found: C, 57.51%; H, 3.96%; N, 17.53%.

*Data for 3-(2-chlorophenyl)-5-methyl-N-(1-methyl-4-(2-(thiophen-2-ylmethylene)hydrazine carbonyl)-1H-pyrazol-5-yl)isoxazole-4-carboxamide* (**6g**). White solid; m.p. 225–227 °C; yield 89.6%; ^1^H-NMR (500 MHz, DMSO-*d*_6_, ppm) *δ*: 11.44 (s, 1H, isoxazole–CONH–), 10.32 (s, 1H, pyrazole–CONH–), 8.54 (s, 1H, –N=CH–), 8.21 (s, 1H, pyrazole–H), 7.98 (s, 1H, Ar–H), 7.65–7.14 (m, 7H, Ar–H, thiophen–H), 3.65 (s, 3H, pyrazole–CH_3_), 2.85 (s, 3H, –CH_3_). ^13^C-NMR (125 MHz, DMSO-*d*_6_, ppm) *δ*: 171.34, 160.60, 158.32, 142.16, 139.72, 137.72, 137.62, 132.80, 132.16, 131.25, 130.75, 130.14, 129.26, 128.85, 128.57, 128.38, 127.96, 113.68, 109.11, 36.64, 12.99. IR (KBr, cm^−1^) *ν*: 3446.79, 3263.56, 2929.87, 1699.29, 1683.86, 1653.00, 1635.64, 1595.13, 1577.77, 1558.48, 1541.12, 1521.84, 1506.41, 1498.69, 1473.62, 1436.97, 1419.61, 1398.39, 1375.25, 1317.38, 1300.02, 1251.80, 1224.80. Anal. Calc. for C_21_H_17_ClN_6_O_3_S: C, 53.79%; H, 3.65%; N, 17.92%; Found: C, 54.08%; H, 4.02%; N, 17.96%.

*Data for 3-(2-chlorophenyl)-N-(4-(2-(2,4-dichlorobenzylidene)hydrazinecarbonyl)-1-methyl-1H- pyrazol-5-yl)-5-methylisoxazole-4-carboxamide* (**6h**). White solid; m.p. 189–190 °C; yield 87.9%; ^1^H-NMR (500 MHz, DMSO-*d*_6_, ppm) *δ*: 11.77 (s, 1H, isoxazole–CONH–), 10.39 (s, 1H, pyrazole–CONH–), 8.67 (s, 1H, –N=CH–), 8.03 (s, 1H, pyrazole–H), 7.80 (d, 1H, *J* = 8.00 Hz, Ar–H), 7.63–7.47 (m, 6H, Ar–H), 3.66 (s, 3H, pyrazole–CH_3_), 2.85 (s, 3H, –CH_3_). ^13^C-NMR (125 MHz, DMSO-*d*_6_, ppm) *δ*: 171.53, 160.76, 158.52, 141.59, 138.08, 137.68, 135.37, 135.20, 134.23, 132.76, 132.19, 132.08, 131.22, 130.17, 129.94, 128.61, 128.53, 127.98, 113.75, 109.32, 108.41, 36.62, 13.00. IR (KBr, cm^−1^) *ν*: 3444.87, 3284.77, 3186.40, 2929.87, 1699.29, 1683.86, 1674.21, 1653.00, 1635.64, 1581.63, 1570.06, 1543.05, 1521.84, 1506.41, 1489.05, 1473.62, 1458.18, 1436.97, 1419.61, 1398.39, 1386.82, 1363.67, 1319.31, 1300.02, 1251.80, 1222.87. Anal. Calc. for C_23_H_17_Cl_3_N_6_O_3_: C, 51.95%; H, 3.22%; N, 15.80%; Found: C, 51.99%; H, 3.48%; N, 15.93%.

*Data for 3-(2-chlorophenyl)-5-methyl-N-(1-methyl-4-(2-(4-nitrobenzylidene)hydrazinecarbonyl)-1H-pyrazol-5-yl)isoxazole-4-carboxamide* (**6i**). White solid; m.p. 155–156 °C; yield 89.7%; ^1^H-NMR (500 MHz, DMSO-*d*_6_, ppm) *δ*: 11.79 (s, 1H, isoxazole–CONH–), 10.37 (s, 1H, pyrazole–CONH–), 8.42 (s, 1H, –N=CH–), 8.31 (s, 1H, pyrazole–H), 8.29 (s, 1H, Ar–H), 8.14–7.92 (m, 2H, Ar–H), 7.61–7.46 (m, 5H, Ar–H), 3.67 (s, 3H, pyrazole–CH_3_), 2.86 (s, 3H, –CH_3_). ^13^C-NMR (125 MHz, DMSO-*d*_6_, ppm) *δ*: 171.53, 160.62, 158.46, 148.23, 144.36, 141.23, 137.77, 132.81, 132.17, 132.09, 130.15, 130.13, 129.40, 128.37, 128.24, 127.96, 127.83, 124.65, 124.56, 113.70, 109.35, 36.66, 12.97. IR (KBr, cm^−1^) *ν*: 3444.87, 3246.20, 3064.89, 2929.87, 1699.29, 1683.86, 1674.21, 1653.00, 1635.64, 1627.92, 1587.42, 1570.06, 1558.48, 1541.12, 1521.84, 1508.33, 1489.05, 1473.62, 1458.18, 1436.97, 1411.89, 1398.39, 1375.25, 1344.38, 1317.38, 1253.73, 1215.15. Anal. Calc. for C_23_H_18_ClN_7_O_5_: C, 54.39%; H, 3.57%; N, 19.30%; Found: C, 54.52%; H, 3.74%; N, 19.36%.

*Data for 3-(2-chlorophenyl)-N-(4-(2-(furan-2-ylmethylene)hydrazinecarbonyl)-1-methyl-1H-pyrazol-5-yl)-5-methylisoxazole-4-carboxamide* (**6j**). White solid; m.p. 147–149 °C; yield 86.4%; ^1^H-NMR (500 MHz, DMSO-*d*_6_, ppm) *δ*: 11.45 (s, 1H, isoxazole–CONH–), 10.36 (s, 1H, pyrazole–CONH–), 8.21 (s, 1H, –N=CH–), 7.85 (s, 1H, pyrazole–H), 7.61–7.47 (m, 5H, Ar-H, furan–H), 6. 91 (t, 1H, *J* = 13.15 Hz, furan–H), 6.63 (s, 1H, furan–H), 3.65 (s, 3H, pyrazole–CH_3_), 2.85 (s, 3H, –CH_3_). ^13^C-NMR (125 MHz, DMSO-*d*_6_, ppm) *δ*: 171.48, 163.12, 160.69, 158.35, 149.97, 145.59, 137.85, 137.60, 136.76, 133.51, 132.81, 132.17, 130.15, 127.96, 127.84, 113.86, 113.72, 112.70, 109.55, 36.63, 12.96. IR (KBr, cm^−1^) *ν*: 3462.22, 3265.49, 2929.87, 1699.29, 1683.86, 1647.12, 1653.00, 1635.64, 1627.92, 1575.84, 1570.06, 1558.48, 1541.12, 1521.84, 1496.76, 1489.05, 1473.62, 1436.97, 1419.61, 1398.39, 1361.74, 1319.31, 1300.02, 1253.73, 1213.23. Anal. Calc. for C_21_H_17_ClN_6_O_4_: C, 55.70%; H, 3.78%; N, 18.56%; Found: C, 55.78%; H, 4.03%; N, 18.89%.

*Data for 3-(2-chlorophenyl)-5-methyl-N-(1-methyl-4-(2-(3,4,5-trimethoxybenzylidene)hydrazine carbonyl)-1H-pyrazol-5-yl)isoxazole-4-carboxamide* (**6k**). White solid; m.p. 206–207 °C; yield 91.2%; ^1^H-NMR (500 MHz, DMSO-*d*_6_, ppm) *δ*: 11.44 (s, 1H, isoxazole–CONH–), 10.28 (s, 1H, pyrazole–CONH–), 8.26 (s, 1H, –N=CH–), 7.80 (s, 1H, pyrazole–H), 7.60–7.47 (m, 4H, Ar–H), 6.99 (s, 1H, Ar–H), 6.97 (s, 1H, Ar–H), 3.83 (s, 6H, –OCH_3_), 3.70 (s, 3H, pyrazole–CH_3_), 3.66 (s, 3H, –OCH_3_), 2.85 (s, 3H, –CH_3_). ^13^C-NMR (125 MHz, DMSO-*d*_6_, ppm) *δ*: 171.58, 161.01, 160.59, 158.45, 158.23, 153.73, 153.71, 147.08, 146.67, 137.70, 136.96, 132.82, 132.17, 130.42, 130.15, 127.95, 127.81, 114.14, 110.70, 109.58, 104.72, 104.44, 60.65, 56.48, 36.68, 13.02. IR (KBr, cm^−1^) *ν*: 3444.87, 3226.91, 31867.40, 3066.82, 2929.87, 1699.29, 1683.86, 1668.43, 1647.21, 1616.35, 1577.77, 1558.48, 1541.12, 1527.62, 1506.41, 1489.05, 1473.62, 1448.54, 1417.68, 1381.03, 1354.03, 1317.38, 1301.95, 1238.30, 1203.58. Anal. Calc. for C_26_H_25_ClN_6_O_6_: C, 56.47%; H, 4.56%; N, 15.20%; Found: C, 56.86%; H, 4.84%; N, 15.47%.

*Data for 3-(2-chlorophenyl)-5-methyl-N-(1-methyl-4-(2-(propan-2-ylidene)hydrazinecarbonyl)-1H-pyrazol-5-yl)isoxazole-4-carboxamide* (**6l**). White solid; m.p. 189–191 °C; yield 87.2%; ^1^H-NMR (500 MHz, DMSO-*d*_6_, ppm) *δ*: 10.16 (s, 1H, isoxazole–CONH–), 9.28 (s, 1H, pyrazole–CONH–), 7.86 (s, 1H, pyrazole–H), 7.60–7.47 (m, 4H, Ar–H), 4.34 (s, 3H, –CH_3_), 3.61 (s, 3H, pyrazole–CH_3_), 2.85 (s, 3H, isoxazole–CH_3_), 2.50 (s, 3H, –CH_3_). ^13^C-NMR (125 MHz, DMSO-*d*_6_, ppm) *δ*: 170.80, 161.37, 159.91, 159.61, 136.57, 135.96, 132.09, 131.43, 131.38, 129.43, 129.38, 127.22, 127.15, 112.97, 108.85, 35.82, 24.84, 18.36, 12.22. IR (KBr, cm^−1^) *ν*: 3444.87, 3066.82, 1699.29, 1683.86, 1668.43, 1653.00, 1635.64, 1616.35, 1577.77, 1558.48, 1541.12, 1521.84, 1506.41, 1489.05, 1458.18, 1473.62, 1429.25, 1411.89, 1386.82, 1338.60, 1313.52, 1279.88, 1236.73. Anal. Calc. for C_19_H_19_ClN_6_O_3_: C, 55.01%; H, 4.62%; N, 20.26%; Found: C, 55.35%; H, 4.98%; N, 20.52%.

*Data for 3-(2-chlorophenyl)-5-methyl-N-(1-methyl-4-(2-(2-methylpropylidene)hydrazinecarbonyl)-1H-pyrazol-5-yl)isoxazole-4-carboxamide* (**6m**). White solid; m.p. 212–214 °C; yield 88.3%; ^1^H-NMR (500 MHz, DMSO-*d*_6_, ppm) *δ*: 11.14 (s, 1H, isoxazole–CONH–), 9.27 (s, 1H, pyrazole–CONH–), 7.85 (s, 1H, pyrazole–H), 7.60–7.47 (m, 5H, –C=NH–, Ar–H), 4.32 (s, 3H, CH_3_), 3.61 (s, 3H, pyrazole–CH_3_), 2.85 (s, 4H, –CH–, –CH_3_), 2.50 (s, 3H, –CH_3_). ^13^C-NMR (125 MHz, DMSO-*d*_6_, ppm) *δ*: 171.15, 166.20, 161.71, 160.25, 136.94, 136.32, 132.43, 131.76, 131.67, 129.74, 127.56, 127.47, 125.47, 119.61, 113.32, 109.20, 36.14, 30.18, 20.12, 12.57. IR (KBr, cm^−1^) *ν*: 3325.28, 3304.06, 3066.82, 1699.29, 1683.86, 1674.21, 1653.00, 1635.64, 1610.56, 1577.77, 1558.48, 1541.12, 1521.84, 1506.41, 1489.05, 1458.18, 1473.62, 1458.18, 1429.25, 1411.89, 1386.82, 1338.60, 1278.81, 1249.87, 1201.65. Anal. Calc. for C_20_H_21_ClN_6_O_3_: C, 56.01%; H, 4.94%; N, 19.60%; Found: C, 56.19%; H, 5.13%; N, 20.01%.

*Data for 3-(2-chlorophenyl)-5-methyl-N-(1-methyl-4-(2-propylidenehydrazinecarbonyl)-1H-pyrazol-5-yl)isoxazole-4-carboxamide* (**6n**). White solid; m.p. 199–201 °C; yield 89.6%; ^1^H-NMR (500 MHz, DMSO-*d*_6_, ppm) *δ:* 11.01 (s, 1H, isoxazole–CONH–), 10.23 (s, 1H, pyrazole–CONH–), 7.91 (s, 1H, pyrazole–H), 7.60–7.39 (m, 5H, –C=NH–, Ar–H), 3.62 (s, 3H, pyrazole–CH_3_), 2.82 (s, 3H, isoxazole–CH_3_), 2.25 (q, 2H, *J* = 14.90 Hz, –CH_2_–), 1.05 (t, 3H, *J* = 14.90 Hz, –CH_3_). ^13^C-NMR (125 MHz, DMSO-*d*_6_, ppm) *δ*: 172.81, 166.66, 161.98, 153.62, 146.78, 138.90, 137.94, 134.20, 133.51, 131.48, 129.28, 126.80, 121.48, 116.59, 38.00, 27.25, 14.25, 12.62. IR (KBr, cm^−1^) *ν*: 3325.28, 3304.06, 3066.82, 1699.29, 1683.86, 1674.21, 1653.00, 1635.64, 1615.63, 1577.77, 1558.48, 1541.12, 1521.84, 1506.41, 1489.05, 1473.62, 1456.26, 1436.97, 1417.68, 1398.39, 1338.60, 1309.31, 1269.81, 1238.74, 1207.29. Anal. Calc. for C_19_H_19_ClN6O_3_: C, 55.01%; H, 4.62%; N, 20.26%; Found: C, 55.36%; H, 4.87%; N, 20.54%.

*Data for 3-(2,6-dichlorophenyl)-N-(4-(2-(4-chlorobenzylidene)hydrazinecarbonyl)-1-methyl-1H-pyrazol-5-yl)-5-methylisoxazole-4-carboxamide* (**6o**). White solid; m.p. 168–170 °C; yield 91.3%; ^1^H-NMR (500 MHz, DMSO-*d*_6_, ppm) *δ*: 11.56 (s, 1H, isoxazole–CONH), 10.26 (s, 1H, pyrazole–CONH), 8.32 (s, 1H, –CH=N–), 8.03–8.01 (d, 1H, *J* = 9.70 Hz, pyrazole–H), 7.74–7.51 (m, 7H, Ar–H), 3.61 (s, 3H, pyrazole–CH_3_), 2.92 (s, 3H, isoxazole–CH_3_). ^13^C NMR (125 MHz, DMSO-*d*_6_, ppm) *δ*: 171.84, 163.47, 159.92, 158.61, 158.40, 145.62, 142.82, 140.37, 137.72, 134.95, 134.91, 133.86, 132.91, 129.47, 129.14, 128.96, 128.93, 127.28, 127.12, 113.60, 109.62, 36.42, 13.17. IR (KBr, cm^−1^) *ν*: 3444.87, 3176.76, 2951.09, 1699.29, 1683.86, 1653.00, 1635.64, 1616.35, 1605.89, 1575.84, 1558.48, 1541.12, 1521.84, 1506.41, 1489.08, 1473.62, 1448.54, 1411.89, 1398.39, 1319.31, 1301.95, 1215.15. Anal. Calc. for C_23_H_17_Cl_3_N_6_O_3_: C, 51.95% H, 3.22%; N, 15.80%; Found: C, 51.98%; H, 3.59; N, 15.96%.

*Data for 3-(2,6-dichlorophenyl)-N-(4-(2-(4-fluorobenzylidene)hydrazinecarbonyl)-1-methyl-1H-pyrazol-5-yl)-5-methylisoxazole-4-carboxamide* (**6p**). White solid; m.p. 154–156 °C; yield 90.6%; ^1^H-NMR (500 MHz, DMSO-*d*_6_, ppm) *δ*: 11.51 (s, 1H, isoxazole–CONH–), 10.25 (s, 1H, pyrazol–CONH–), 8.33 (s, 1H, –CH=N–), 8.05 (d, 1H, *J* = 18.35 Hz, pyrazole–H), 7.78–7.57 (m, 5H, Ar–H), 7.31 (t, 2H, *J* = 17.75 Hz, Ar–H), 3.62 (s, 3H, pyrazole–CH_3_), 2.92 (s, 3H, isoxazole–CH_3_). ^13^C-NMR (125 MHz, DMSO-*d*_6_, ppm) *δ*: 171.60, 171.21, 163.90, 162.81, 161.93, 159.29, 157.87 (d, 1C, 4-F-Ph-C), 145.21, 142.34, 139.74, 138.51, 137.08, 134.29, 132.26, 130.87, 129.00, 128.30, 126.66, 115.88, 115.71, 112.96, 109.02, 107.44, 35.81, 12.54. IR (KBr, cm^−1^) *ν*: 3446.79, 3068.75, 2951.09, 1699.29, 1683.86, 1653.00, 1635.64, 1627.92, 1602.85, 1575.84, 1558.48, 1541.12, 1521.84, 1506.41, 1489.08, 1473.62, 1448.54, 1419.61, 1398.39, 1319.31, 1298.09, 1234.44, 1213.23. Anal. Calc. for C_23_H_17_Cl_2_FN_6_O_3_: C, 53.61%; H, 3.33%; N, 16.31%; Found: C, 53.69%; H, 3.75%; N, 16.51%.

*Data for 3-(2,6-dichlorophenyl)-N-(4-(2-(furan-2-ylmethylene)hydrazinecarbonyl)-1-methyl-1H-pyrazol-5-yl)-5-methylisoxazole-4-carboxamide* (**6q**). White solid; m.p. 255–257 °C; yield 86.5%; ^1^H-NMR (500 MHz, DMSO-*d*_6_, ppm) *δ*: 11.46 (s, 1H, isoxazole–CONH–), 10.29 (s, 1H, pyrazole–CONH–), 8.22 (s, 1H, –CH=N–), 7.99 (d, 1H, pyrazole–H), 7.85 (s, 1H, Ar–H), 7.64–7.52 (m, 3H, Ar–H, pyrazole–H, furan–H), 6.91 (d, 1H, *J* = 9.75 Hz, furan–H), 6.63 (s, 1H, furan–H), 3.62 (s, 3H, pyrazole–CH_3_), 2.93 (s, 3H, isoxazole–CH_3_). ^13^C-NMR (125 MHz, DMSO-*d*_6_, ppm) *δ*: 171.82, 163.26, 159.95, 158.32, 149.98, 145.59, 140.80, 139.31, 137.64, 136.81, 134.92, 133.62, 132.90, 128.94, 127.28, 113.83, 112.70, 109.67, 107.80, 36.44, 13.16. IR (KBr, cm^−1^) *ν*: 3446.79, 3068.75, 2953.02, 1689.64, 1653.00, 1635.64, 1602.85, 1558.48, 1541.12, 1521.84, 1506.41, 1489.08, 1473.62, 1431.18, 1409.96, 1398.39, 1330.88, 1317.38, 1255.66, 1220.94. Anal. Calc. for C_21_H_16_Cl_2_N_6_O_4_: C, 51.76%; H, 3.31%; N, 17.25%; Found: C, 51.84%; H, 3.36%; N, 17.28%.

*Data for 3-(2,6-dichlorophenyl)-5-methyl-N-(1-methyl-4-(2-(thiophen-2-ylmethylene)hydrazinecarbonyl) -1H-pyrazol-5-yl)isoxazole-4-carboxamide* (**6r**). White solid; m.p. 248–251 ^o^C; yield 85.9%; ^1^H-NMR (500 MHz, DMSO-*d*_6_, ppm) *δ*: 11.43 (s, 1H, isoxazole–CONH–), 10.22 (s, 1H, pyrazole–CONH–), 8.54 (s, 1H, –CH=N–), 7.98 (s, 1H, pyrazole–H), 7.65–7.49 (m, 5H, Ar–H, thiophen–H), 7.13 (s, 1H, thiophen–H), 3.61(s, 3H, pyrazole–CH_3_), 2.91 (s, 3H, isoxazole–CH_3_). ^13^C-NMR (125 MHz, DMSO-*d*_6_, ppm) *δ*: 172.35, 171.82, 163.26, 159.95, 158.64, 158.45, 158.32, 149.98, 149.87, 145.59, 140.80, 139.31, 137.69, 137.64, 136.81, 134.92, 133.62, 132.90, 128.94, 128.64, 127.28, 113.83, 113.61, 113.36, 112.70, 109.67, 107.80, 36.72, 13.16. IR (KBr, cm^−1^) *ν*: 3446.79, 3265.49, 2953.02, 1699.29, 1683.86, 1653.00, 1635.64, 1595.13, 1575.84, 1558.48, 1541.12, 1521.84, 1506.41, 1489.08, 1473.62, 1458.18, 1436.97, 1398.39, 1373.32, 1315.45, 1294.24, 1251.80, 1222.87. Anal. Calc. for C_21_H_16_Cl_2_N_6_O_3_S: C, 50.11%; H, 3.20%; N, 16.70%; Found: C, 50.28%; H, 3.43%; N, 16.76%.

### 2.2. Biological Evaluations

Table 1 shows that most of the title compounds exhibited good antiviral activity against TMV in vivo. Especially, among the title compounds evaluated, compounds **6a** and **6h** exhibited good curative activity against TMV, with inhibition rates of 56.2% and 55.6% at 500 μg/mL, respectively, which were similar to that of Ningnanmycin (54.6%). Meanwhile, compounds **6c**, **6d**, **6n**, and **6k** exhibited significant protection activity against TMV, with inhibition rates of 66.2%, 64.5%,64.2%, and 64.7% at 500 μg/mL, respectively, which were equal to that of Ningnanmycin (63.8%). In addition, compounds **6c**, **6o**, **6p**, **6q**, and **6r** revealed excellent inactivation activities against TMV, with inhibition rates of 91.4%, 92.3%, 91.7%, 93.2%, and 92.6% at 500 μg/mL, respectively, which were similar to that of Ningnanmycin (92.5%).

As shown in Table 2, compounds **6a**, **6h** and **6k** displayed excellent curative activities against TMV, with the 50% effective concentration (EC_50_) values of 240.8, 255.4 and 267.4 μg/mL, which were even better than that of Ningnanmycin (286.4 μg/mL). Meanwhile, as can be seen from Table 2, compounds **6c**, **6d**, **6h** and **6j** demonstrated excellent protection activities against TMV, with the EC_50_ values of 148.4, 184.9, 189.7 and 176.5 μg/mL, which were superior to that of Ningnanmycin (198.2 μg/mL). Furthermore, compounds **6q** and **6r**, as shown in Table 2, exhibited the best inactivation activity, with the EC_50_ values of 37.3 and 45.1 μg/mL, which were even better than that of Ningnanmycin (46.3 μg/mL). 

As an extension of this approach, the structure-activity relationships (SAR) were analyzed on the basis of the activity values in Table 1 and Table 2. First, when R^1^ and R^2^ substituent groups were 2-Cl and H, respectively, R^3^ was substituted with 4-Cl-Ph, 2,4-diCl-Ph, and 3,4,5-triMe-Ph groups, the corresponding target compounds **6a**, **6h** and **6k** exhibited excellent curative activity against TMV, which was superior to those of Ningnanmycin and the other target compounds. Second, when R^1^ and R^2^ were substituted with 2-Cl and H groups, respectively, R^3^ was 2-Br-Ph, 2-Cl-Ph, 2,4-diCl-Ph, and Furyl groups, the corresponding target compounds **6c**, **6d**, **6h** and **6j** exhibited remarkable protection activity against TMV than those of Ningnanmycin and the other target compounds. Third, when R^1^, R^2^, and R^3^ substituent groups were 2,6-diCl, H, and Furyl, the corresponding compound **6q** revealed the best inactivation activity which was even better than those of Ningnanmycin and the other target compounds. Finally, as shown in Table 1, compound **6** showed better anti-TMV activity than those of compound **3**. Therefore, we found that, when the presence of the –Cl at 2-position or 2,6-positions of phenyl at R^1^ substituent group, the presence of small groups (–H) at R^2^ substituent group, the corresponding compounds presented good antiviral activities. We also found that introducing a hydrazine moiety into the target compounds could enhance the anti-TMV activity, which were also observed in previous studies of Li et al. and Yang [20,24].

## 3. Experimental

### 3.1. General Methods

The melting points of the products were determined on a XT-4 binocular microscope (Beijing Tech Instrument Co., Beijing, China) and were not corrected. The IR spectra were recorded on a Bruker VECTOR 22 spectrometer (Bruker Optics Inc. Billerica, MA, USA) in KBr disk. ^1^H-NMR and ^13^C-NMR (solvent DMSO-*d*_6_) spectral analyses were performed on a JEOL-ECX 500 NMR spectrometer (JEOL Ltd., Tokyo,Japan) at room temperature using tetramethylsilane (TMS) as an internal standard. Elemental analysis was performed on an Elementar Vario-III CHN analyser (Elementar, Frankfurt, Germany). Microwave experiments were carried out using a CEM Discover Labmate microwave apparatus (Discover^®^LabMate instrument, CEM Corporate Matthews, NC, USA). Analytical TLC was performed on silica gel GF_254_ (200–300 mesh). Column chromatographic purification was carried out using silica gel. Commercial reagents were used as received, unless otherwise indicated. All solvents were dried by standard methods in advance and distilled before use.

### 3.2. General Procedure for the Preparation of the Intermediate 5

As shown in Scheme 1, 3-substituted phenyl-5-methylisoxazole-4-carboxylic acid (0.02 mol) was added to distillated SOCl_2_ (50 mL) and reacted at 80 °C for 5 h to obtain intermediate **2**. Then, intermediate **2** (0.02 mol) was added dropwise to a stirred solution of 5-amino-1-methyl-1*H*-pyrazole-4-carboxylic acid (0.02 mol) in anhydrous tetrahydrofuran (THF, 100 mL) and trimethylamine, the reaction mixture was stirred at room temperature for 2.0 h. The reaction mixture was poured into cold 5.0% dilute HCl solution (200 mL). The solid obtained was filtered, washed several times with water, dried to give the crude product which was further recrystallized and dried to give intermediate **3**. A mixture of intermediate **3** (0.02 mol) and acetic anhydride (0.2 mol) was heated under reflux for 4.0 h. The solvent was removed under reduced pressure. The residue was washed with water. The separated solid was collected by filtration, washed with water, dried, and recrystallized and dried to give intermediate **4**. To a solution of intermediate **4** (10 mmol) in THF (50 mL), 6 mL of 80% hydrazine hydrate was added slowly at room temperature. Then, the mixture was further reacted at room temperature for 2 h. The solvent was removed under reduced pressure, and the residue was washed with water and anhydrous ethanol to give the crude product, then recrystallized by ethanol and dried under vacuum to give the key intermediate 3-(2-chlorophenyl)-*N*-(4-(hydrazinecarbonyl)-1-methyl-1*H*-pyrazol-5-yl)-5-methyl isoxazole-4-carboxamide **5**.

### 3.3. General Procedure for the Preparation of the Target Compounds 6a–6r

As shown in Scheme 1, different aldehydes and ketones (1 mmol) were added to a well-stirred solution of **5** (1 mmol) in ethanol (8 mL), and then a few drops of glacial acetic acid were added. The resulting mixture was refluxed for 2 h to afford a solid, and then filtered and recrystallized from a mixture of ethanol and DMF (1:1 in volume) to gain the title compounds **6a**–**6r**.

### 3.4. In Vivo Antiviral Activity Test

#### 3.4.1. Purification of TMV

Using the Gooding method [37], the virus was multiplied in *N. tabacum* cv. K326 and purified. The absorbance values were estimated at 260 nm using an ultraviolet spectrophotometer. The virus concentration was calculated by the following formula, where *E* represents the extinction coefficient for TMV, *E*^0.1%^_1cm_^260nm^ is 3.1:

Virus concentration (mg/mL) = (A_260_ × dilution ratio)/*E*^0.1%^_1cm_^260nm^

#### 3.4.2. Protection Activity of the Title Compounds against TMV in Vivo

The compound solution was smeared on the left side of growing *N. tabacum* L. leaves of the same age. The solvent without compound solution was smeared on the right side of the leaves to serve as the control. The leaves, which were previously scattered with silicon carbide, were inoculated with the virus after 12 h using a brush dipped in 6 × 10^−3^ mg/mL TMV and subsequently washed with water and rubbed softly along the nervature once or twice. After three to four days of the inoculation, the local lesions were counted. Three replications were reproduced for each compound.

#### 3.4.3. Curative Activity of the Title Compounds against TMV in Vivo

The leaves were inoculated with TMV (concentration of 6 × 10^−3^ mg/mL) by dipping and brushing the whole leaves, which were previously scattered with silicon carbide. The leaves were then washed with water after inoculation for 0.5 h. The compound solution was smeared on the left side of the leaves, and the solvent without compound solution was smeared on the right side for control. The number of local lesions was counted and recorded three to four days after the inoculation. Three replications were reproduced for each compound.

#### 3.4.4. Inactivation Activity of the Title Compounds against TMV in Vivo

The virus was inhibited after it was mixed with a compound solution of the same volume for 30 min. The right side of the *N. tabacum* L. leaves was then inoculated with the solvent and virus mixture for control. All of the leaves were previously scattered with silicon carbide. The number of local lesions was recorded three to four days after the inoculation. Three replications were reproduced for each compound.

The inhibition rates of the compounds were calculated according to the following formula:

Inhibition rate (%) = [(average local lesion number of control (not treated with compounds) − average local lesion number smeared with compounds)/average local lesion number of control (not treated with compounds)] × 100%

## 4. Conclusions

In summary, a series of novel pyrazole-hydrazone derivatives containing an isoxazole moiety were obtained with moderate yields and their antiviral activities against TMV were examined by a half-leaf method. Bioassay results revealed that some of the target compounds exhibited better antiviral activity against TMV in vivo than Ningnanmycin. Among the title compounds, compound **6a** exhibited better curative activity against TMV than that of Ningnanmycin. Meanwhile, compound **6c** showed remarkable protection activity against TMV, which was superior to that of Ningnanmycin. Moreover, compound **6q** exhibited better inactivation activity against TMV which were superior to that of Ningnanmycin. The antibacterial tests showed that introducing a hydrazine moiety into the target compounds could enhance the anti-TMV activity; meanwhile, when the presence of the –Cl at 2-position or 2,6-positions of phenyl in the R^1^ substituent group, the presence of small groups (–H) in the R^2^ substituent group, the corresponding compounds presented good antiviral activities. Therefore, this study demonstrated that this series of pyrazole-hydrazone derivatives containing an isoxazole moiety can be considered for further development as a new class of tobacco protection agents. 

## Figures and Tables

**Table 1:** Antiviral activity of the title compounds against TMV at 500 μg/mL in vivo.

Compounds	Curative Activity (%) ^a^	Protection Activity (%) ^a^	Inactivation Activity (%) ^a^
**3a**	20.6 ± 3.3	30.1 ± 2.3	42.7 ± 3.2
**3b**	21.5 ± 1.9	28.9 ± 3.0	39.4 ± 2.2
**6a**	56.2 ± 1.3	60.3 ± 2.1	73.3 ± 1.4
**6b**	48.3 ± 1.5	56.5 ± 3.2	81.5 ± 2.5
**6c**	51.4 ± 2.1	66.2 ± 2.5	91.4 ± 2.0
**6d**	47.1 ± 1.4	64.5 ± 1.7	78.2 ± 2.1
**6e**	44.4 ± 1.7	45.6 ± 1.2	61.4 ± 2.0
**6f**	47.7 ± 2.7	59.3 ± 1.1	71.8 ± 2.1
**6g**	50.9 ± 3.3	47.8 ± 3.4	74.1 ± 1.5
**6h**	55.6 ± 2.0	64.2 ± 1.9	84.9 ± 2.6
**6i**	43.3 ± 3.1	55.3 ± 2.8	73.6 ± 2.1
**6j**	52.8 ± 2.2	39.4 ± 2.4	65.9 ± 2.2
**6k**	51.3 ± 2.6	64.7 ± 2.9	81.5 ± 2.3
**6l**	54.7 ± 2.1	56.9 ± 1.2	66.7 ± 3.2
**6m**	33.6 ± 2.4	40.7 ± 1.8	54.3 ± 2.1
**6n**	27.5 ± 2.5	35.8 ± 2.3	46.5 ± 2.6
**6o**	23.3 ± 1.1	56.2 ± 2.3	92.3 ± 1.8
**6p**	40.7 ± 3.2	49.3 ± 3.1	91.7 ± 1.5
**6q**	21.9 ± 2.2	36.3±1.0	93.2 ± 1.9
**6r**	48.9±2.0	62.6 ± 1.2	92.6 ± 2.2
Ningnanmycin	54.6±1.6	63.8 ± 2.9	92.5 ± 1.3

^a^ Average of three replicates.

**Table 2:** The EC_50_ values of the testing compounds against TMV in vivo.

Compounds	EC_50_ (μg/mL) ^a^		
	Curative Activity	Protection Activity	Inactivation Activity
**6a**	240.8 ± 2.2	287.4 ± 2.3	172.3 ± 2.1
**6b**	457.4 ± 3.4	369.6 ± 3.2	102.0 ± 1.9
**6c**	342.6 ± 4.3	148.4 ± 3.3	62.2 ± 1.6
**6d**	516.9 ± 2.5	184.9 ± 2.0	128.3 ± 2.7
**6f**	505.8 ± 3.3	323.8 ± 3.5	194.6 ± 2.8
**6g**	362.4 ± 1.9	/	150.9 ± 4.0
**6h**	255.4 ± 2.3	189.7 ± 2.7	83.9 ± 3.1
**6i**	315.2 ± 3.6	/	/
**6j**	343.7 ± 4.2	176.5 ± 2.8	98.0 ± 2.6
**6k**	267.4 ± 3.1	342.5 ± 3.6	247.4 ± 4.5
**6o**	/	381.8 ± 3.6	56.4 ± 1.7
**6p**	632.3 ± 5.3	509.5 ± 3.2	64.2 ± 2.3
**6q**	/	/	37.3 ± 1.9
**6r**	455.3 ± 4.6	224.8 ± 1.9	45.1 ± 2.4
Ningnanmycin	286.4 ± 2.2	198.2 ± 2.1	46.3 ± 1.9

^a^ Average of three replicates.

## Supplementary Materials

The ^1^H-NMR and ^13^C-NMR for all the synthesized compounds are available online.
