# Supplementary material for: Novel Pyrazole-Hydrazone Derivatives Containing an Isoxazole Moiety: Design, Synthesis, and Antiviral Activity
Source: Molecules. 2018 Jul 20;23(7):1798. doi: 10.3390/molecules23071798 (PMC6100116; doi:10.3390/molecules23071798)
Supplement: Supplementary file 1 [file molecules-23-01798-s001.pdf]

Article

Novel Pyrazole-Hydrazone Derivatives Containing an Isoxazole Moiety: Design, Synthesis, and Antiviral Activity

Zaibo Yang [1,*], Pei Li [2] and Xiuhai Gan [3]

[1] School of Chemistry and Chemical Engineering, Qiannan Normal University for Nationalities, Guizhou, Duyun 558000, China
[2] Qiandongnan Engineering and Technology Research Center for Comprehensive Utilization of National Medicine, Kaili University, Guizhou, Kaili 556011, China; pl19890627@126.com
[3] College of Chemistry and Life Science, Guizhou Normal College, Guiyang 550018, China; gxh200719@163.com
* Correspondence: qnsyyzb27001@sgmtu.edu.cn; Tel.: 86-85-4873-7046

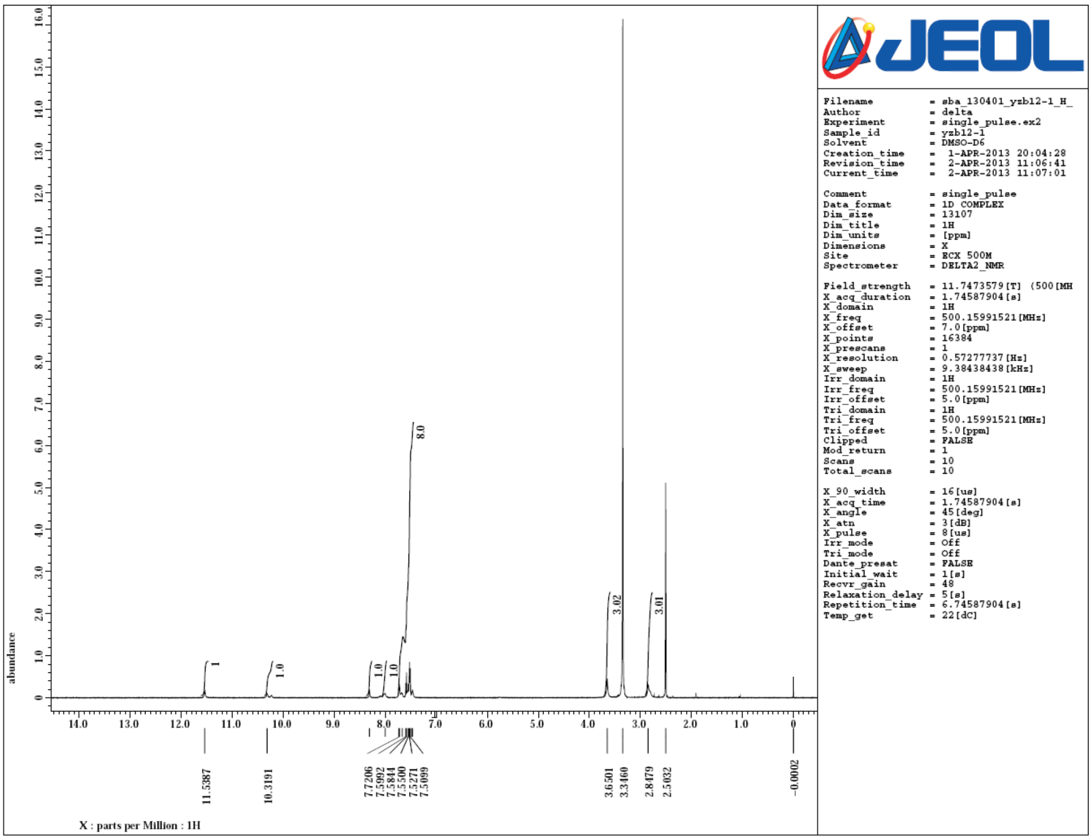

^{1}H NMR for compound **6a.**

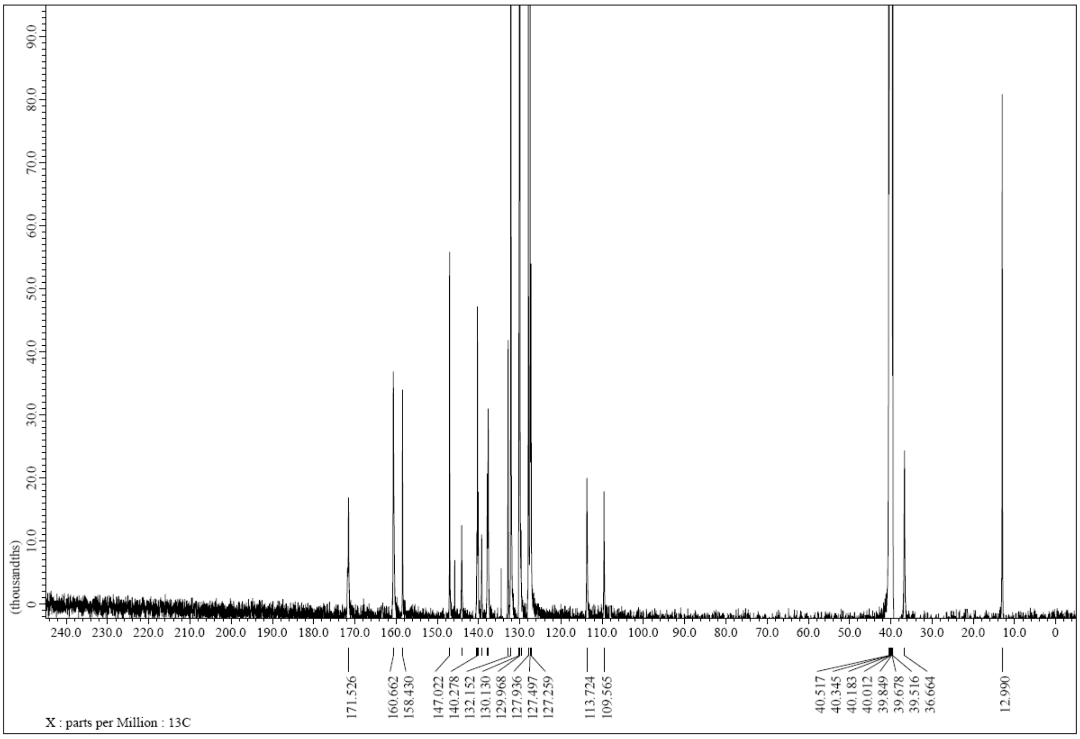

^{13}C NMR for compound **6a.**

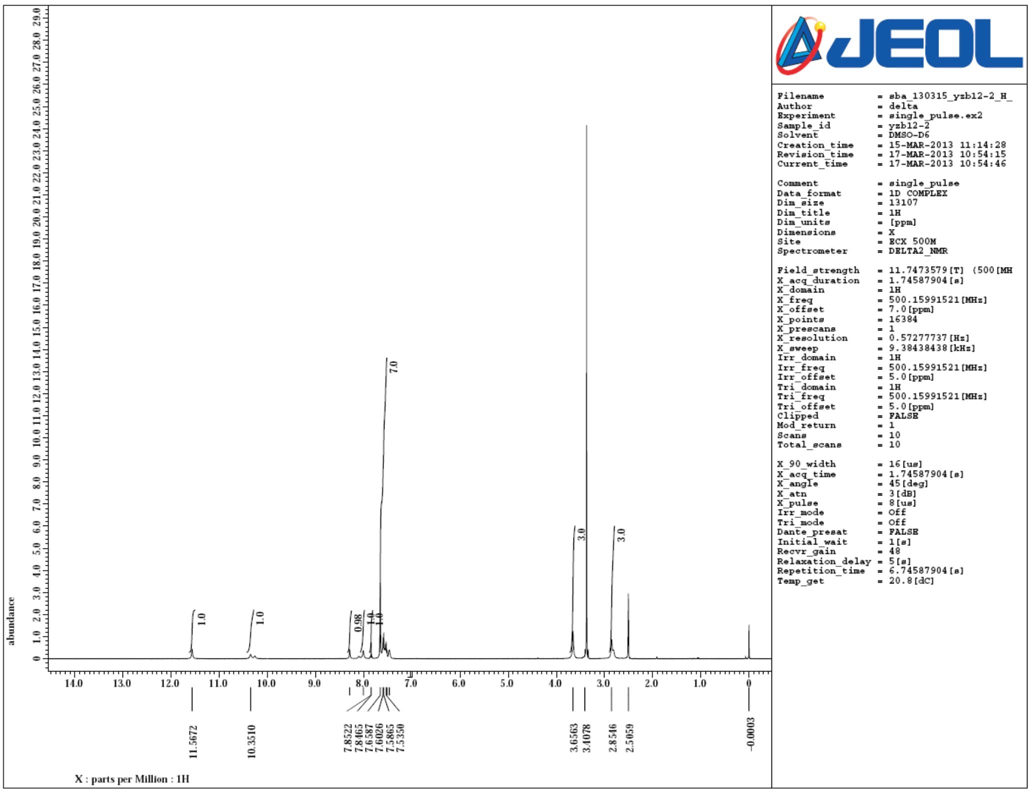

^{1}H NMR for compound **6b**

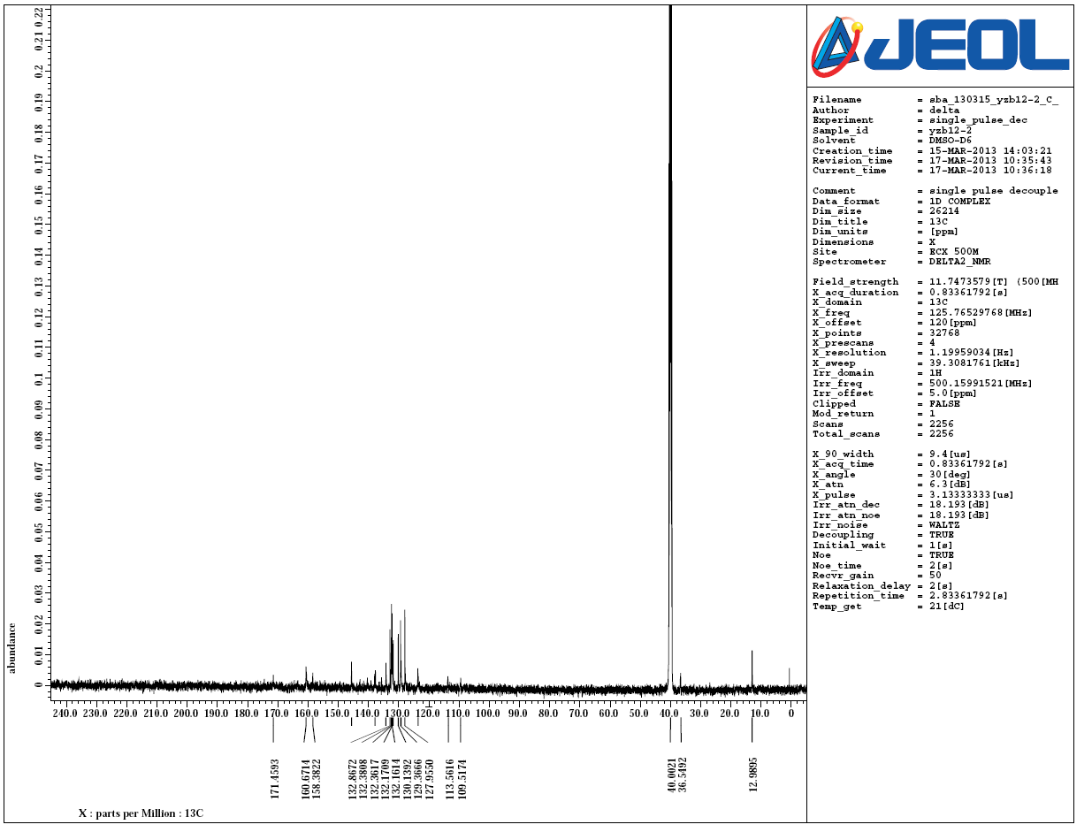

^{13}C NMR for compound **6b**

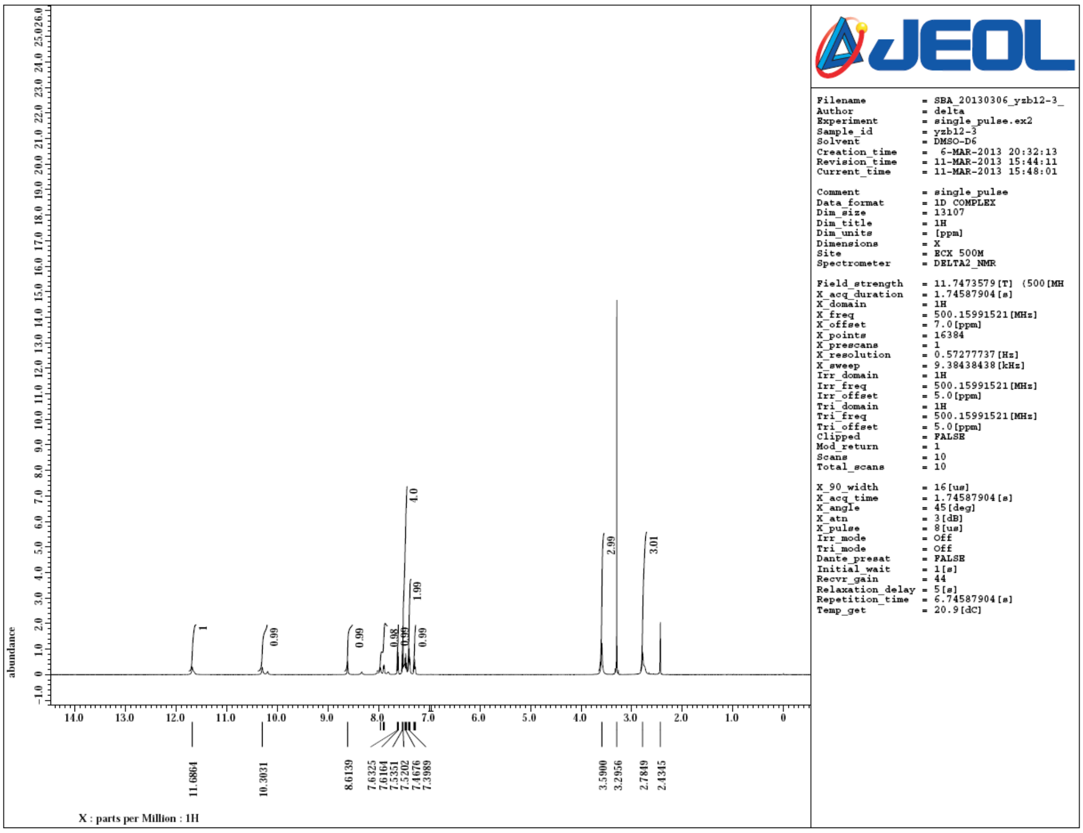

^{1}H NMR for compound **6c**

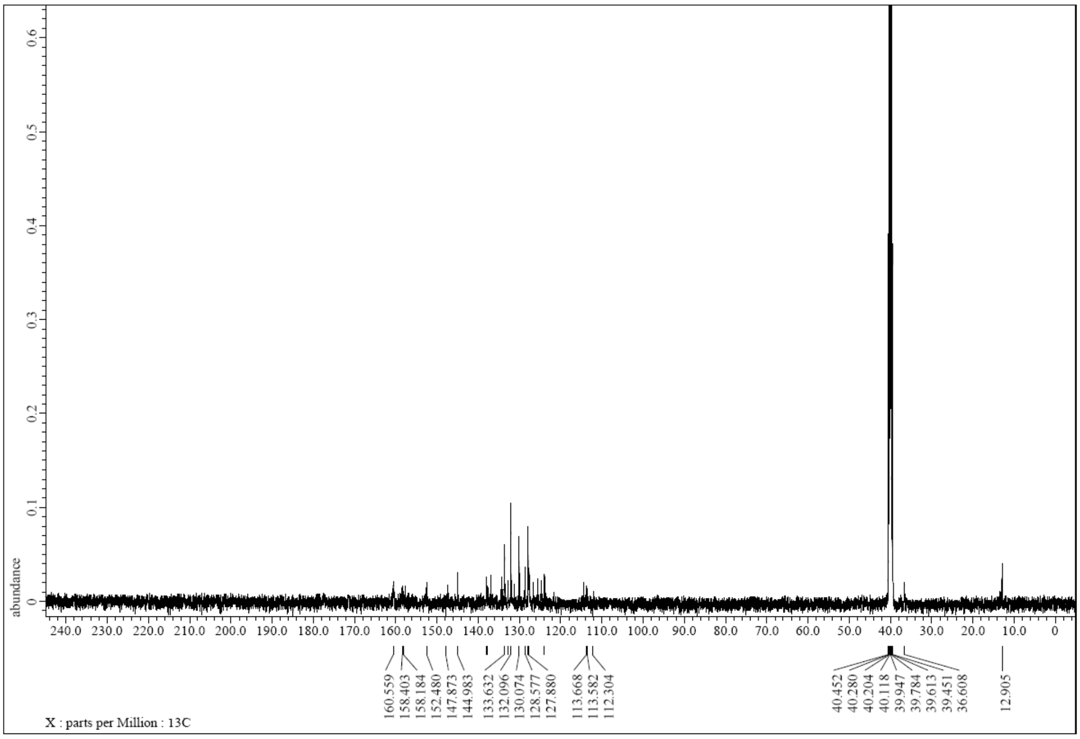

^{13}C NMR for compound **6c**

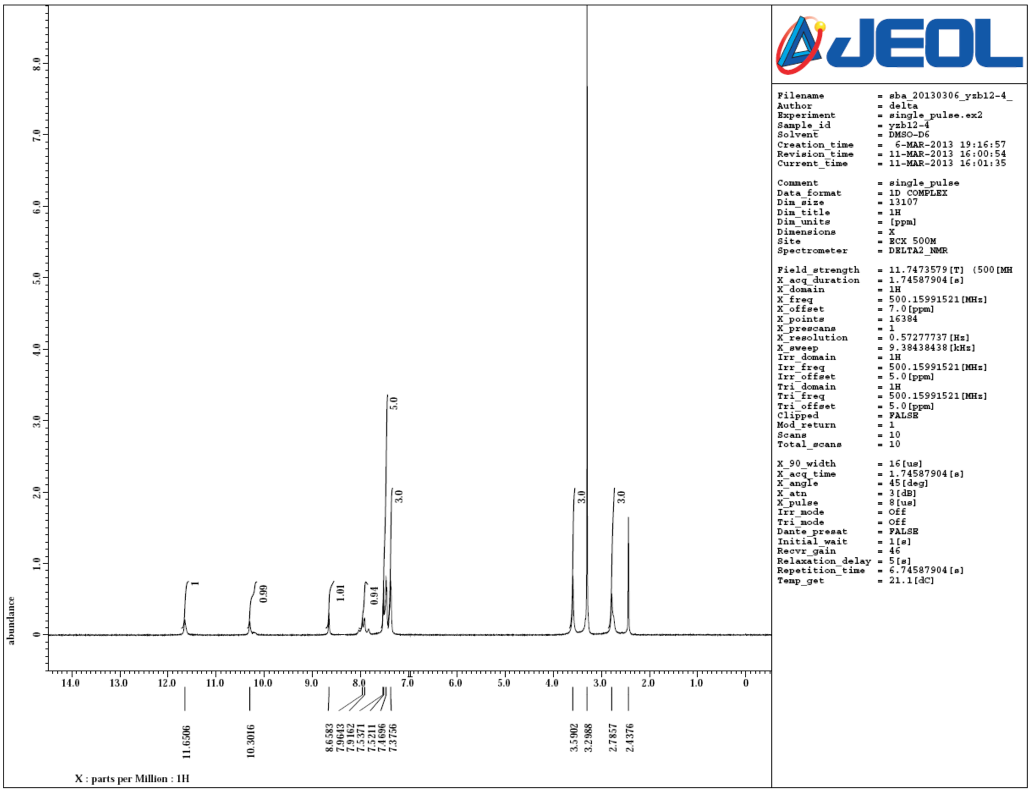

^{1}H NMR for compound **6d**

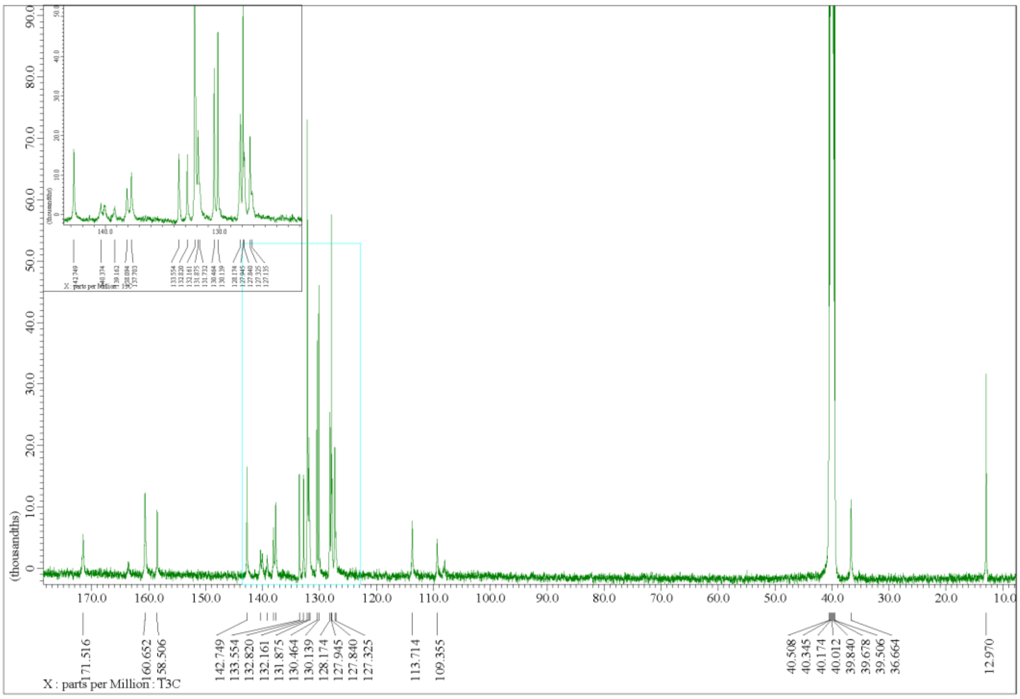

^{13}C NMR for compound **6d**

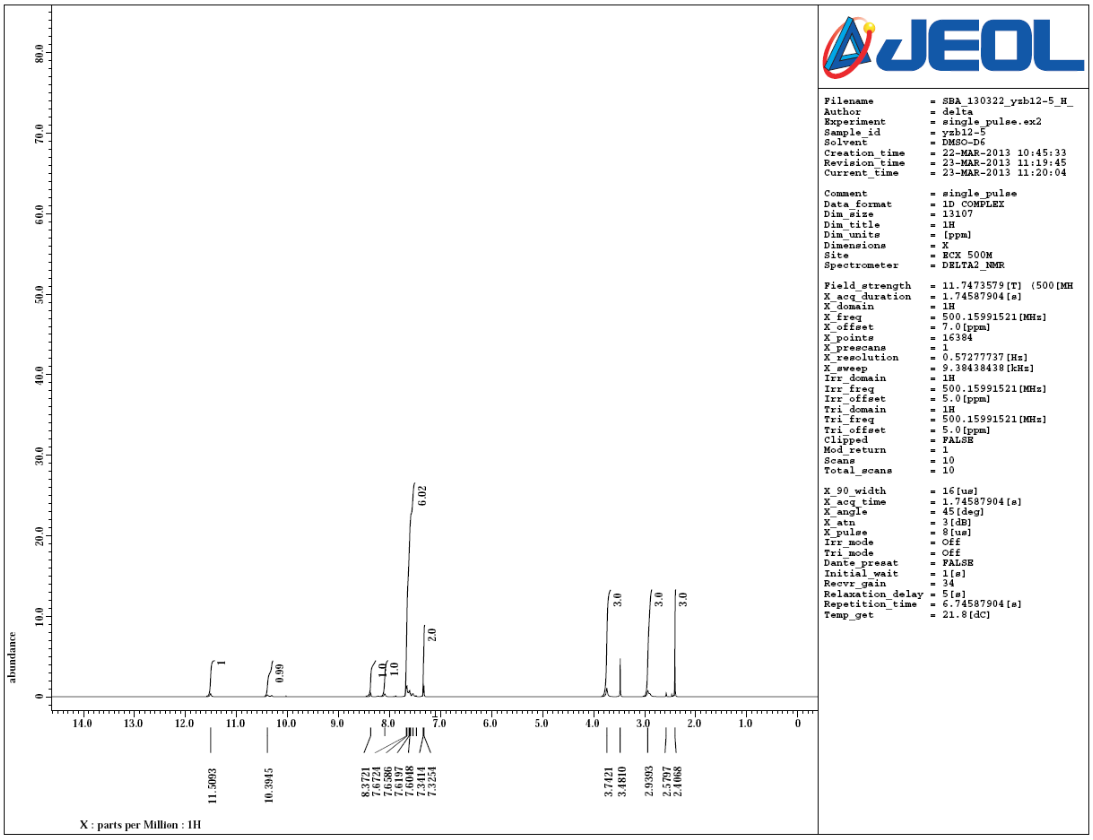

^{1}H NMR for compound **6e**

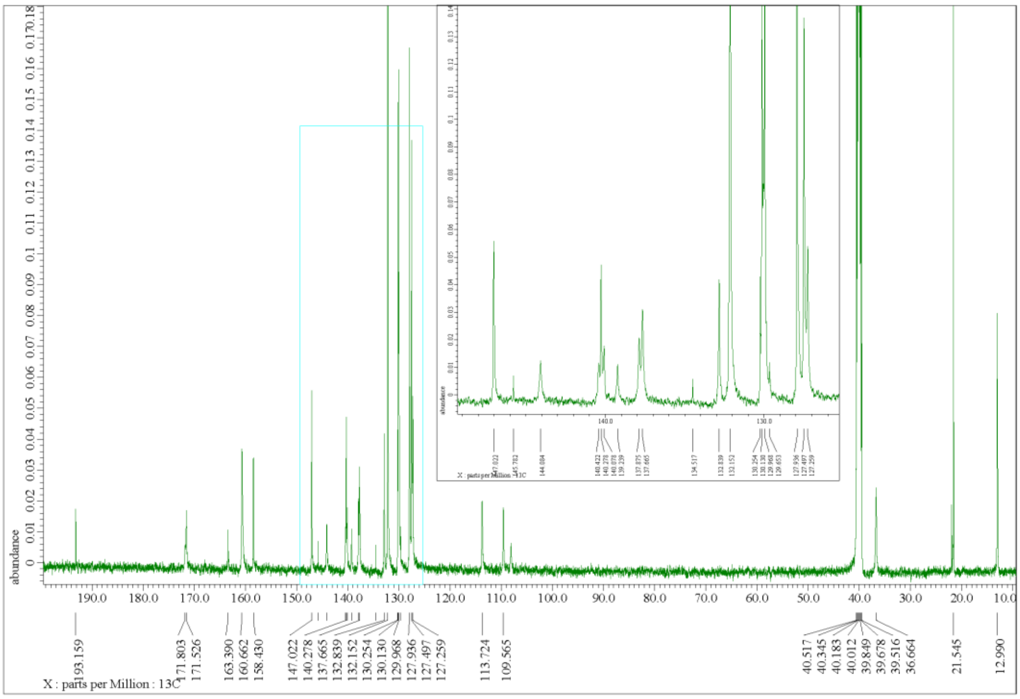

^{13}C NMR for compound **6e**

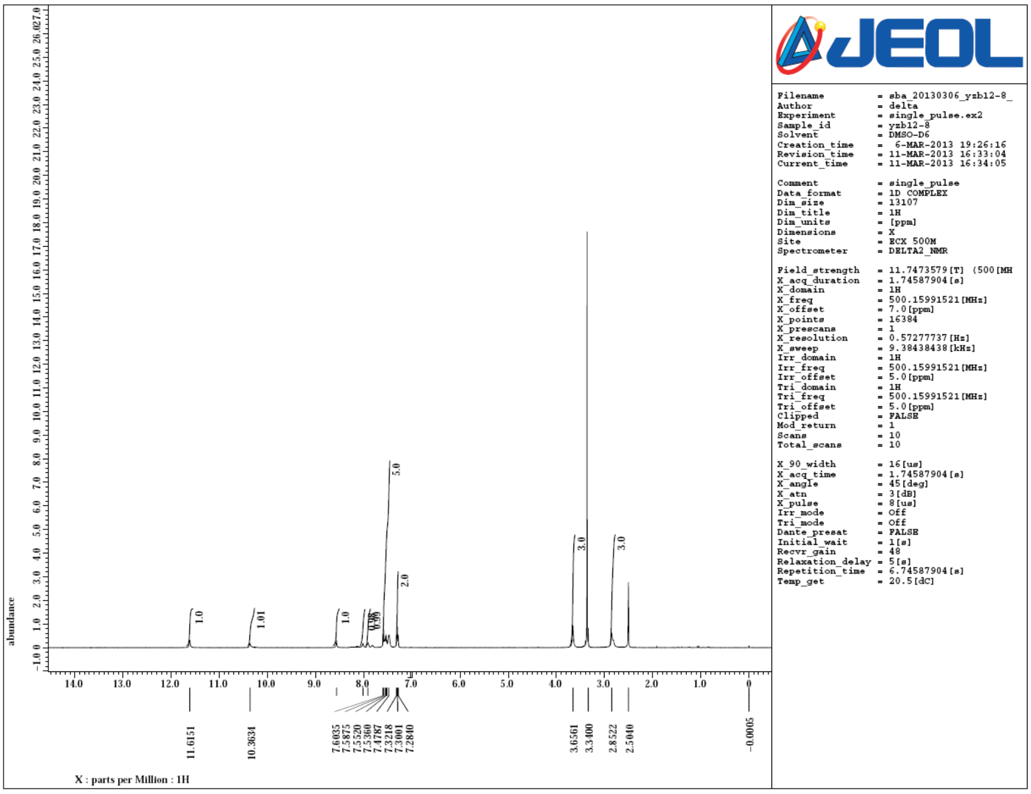

^{1}H NMR for compound **6f**

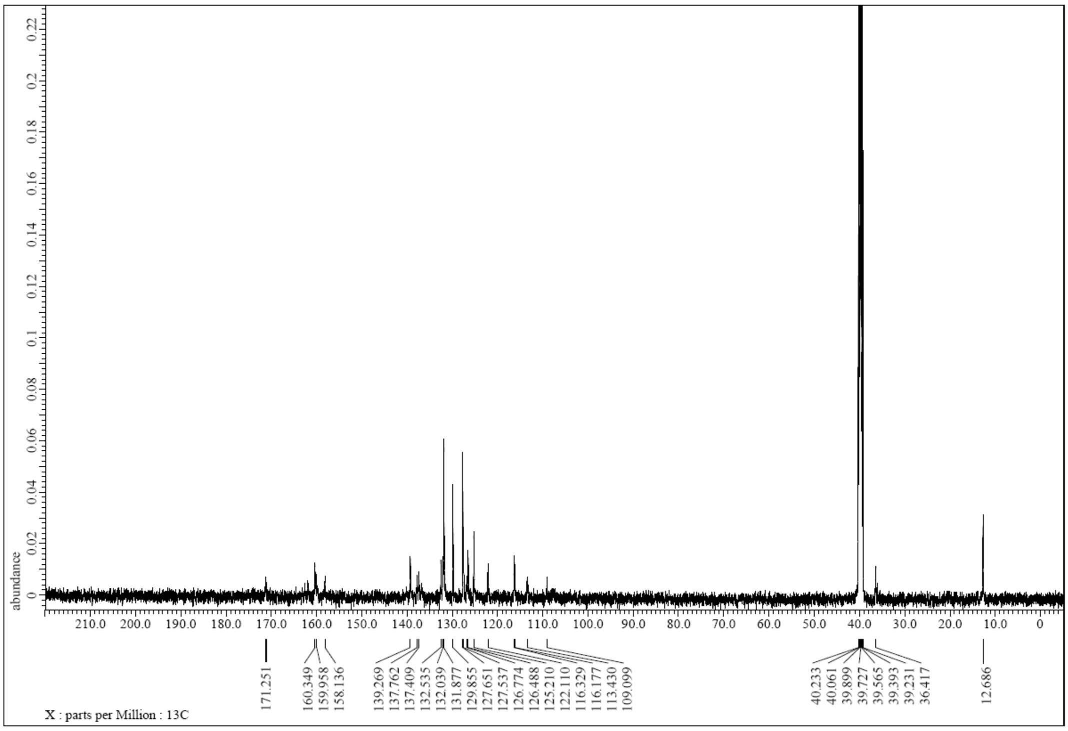

^{13}C NMR for compound **6f**

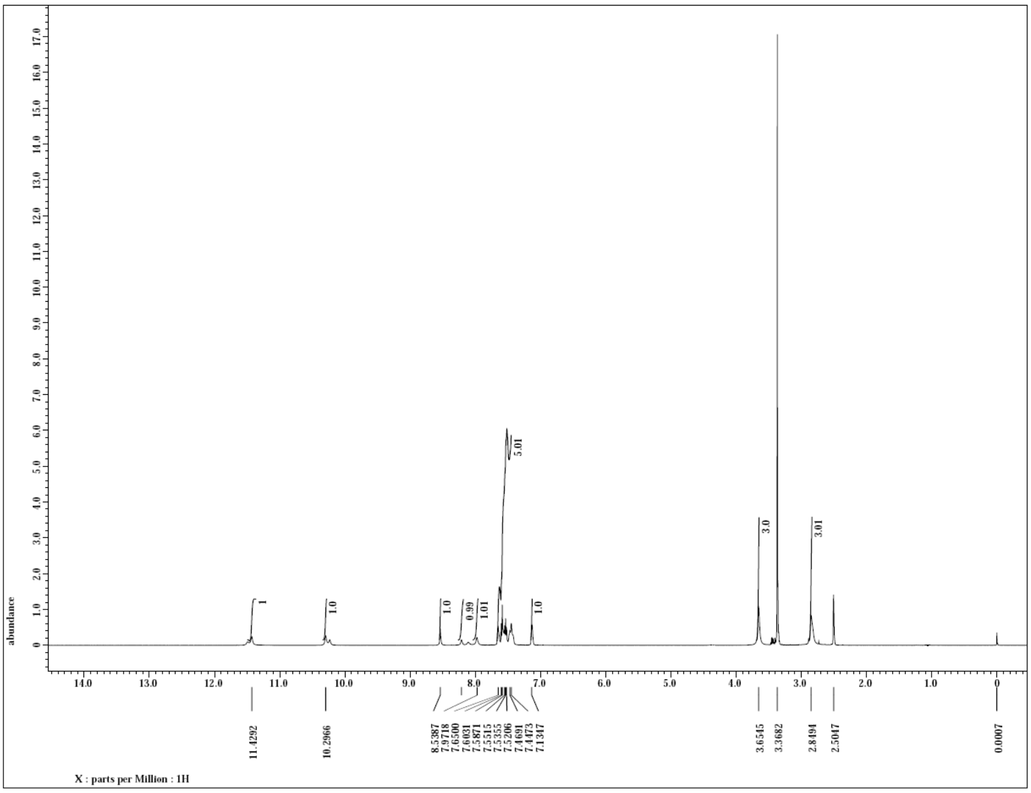

^{1}H NMR for compound **6g**

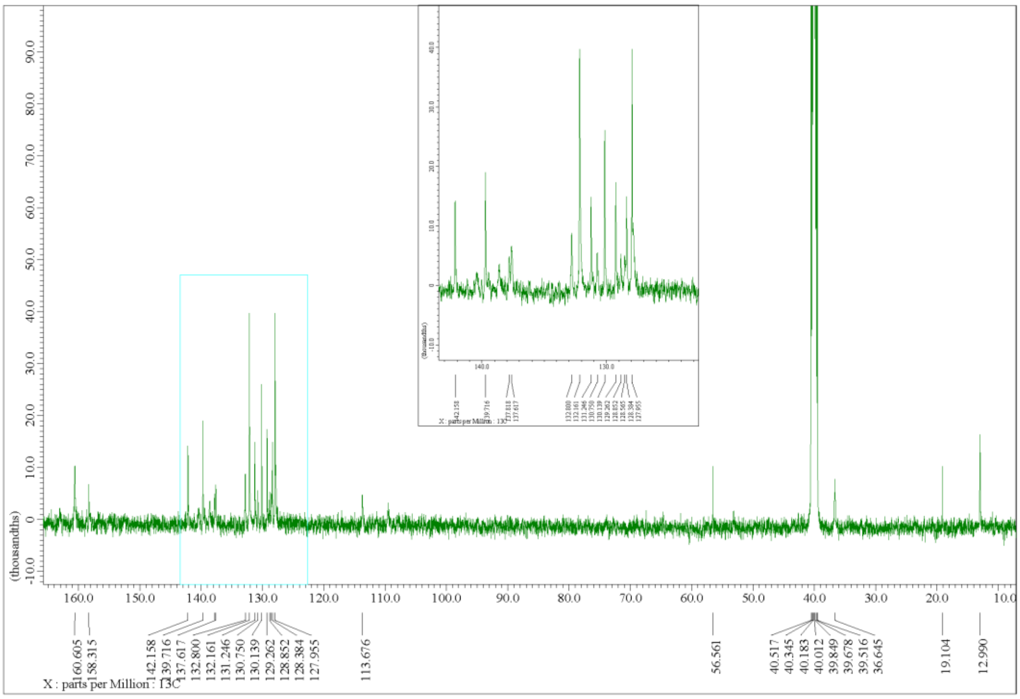

^{13}C NMR for compound **6g**

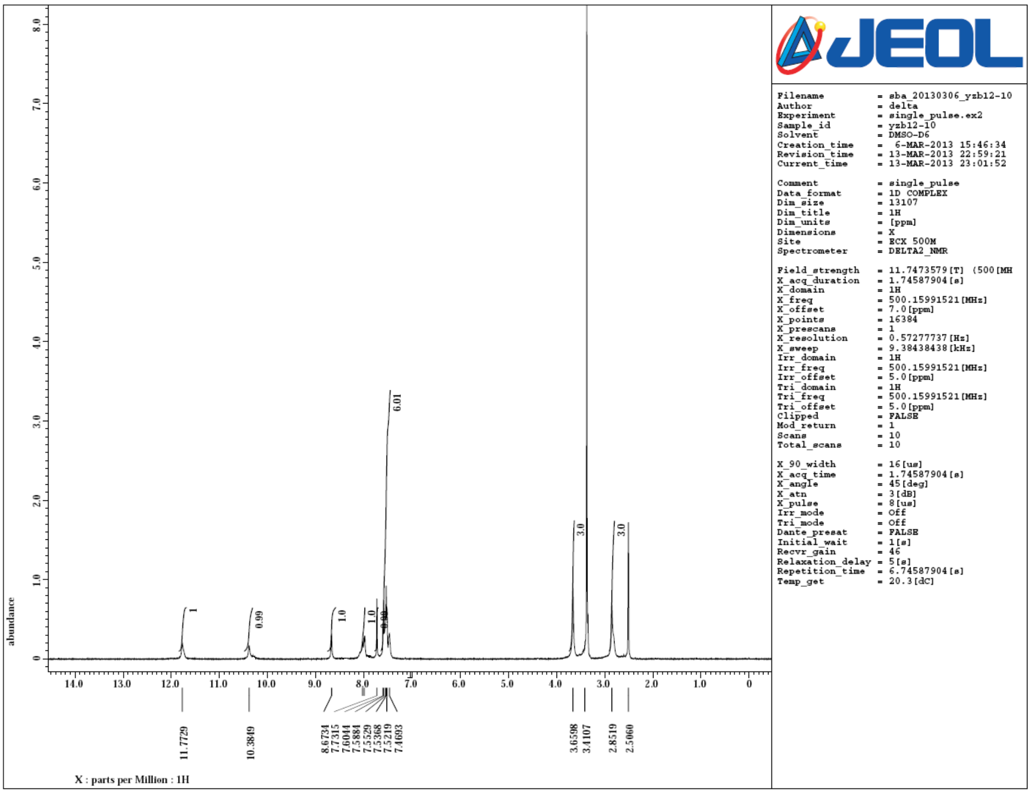

^{1}H NMR for compound **6h**

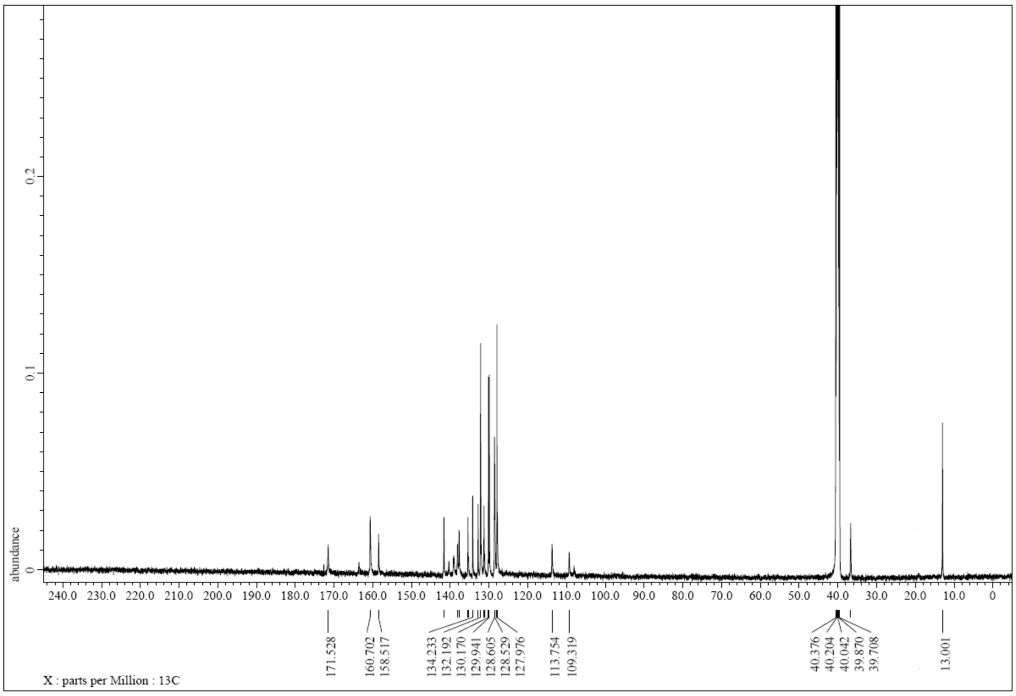

^{13}C NMR for compound **6h**

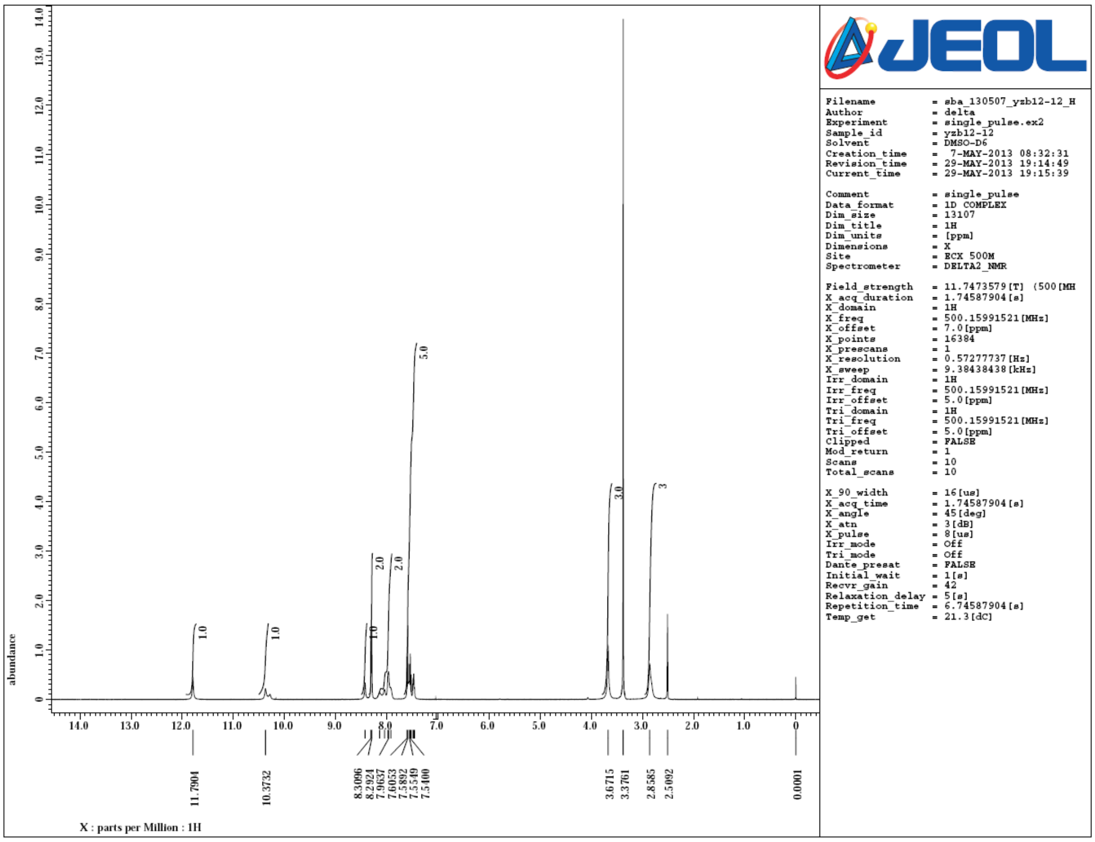

^{1}H NMR for compound **6i**

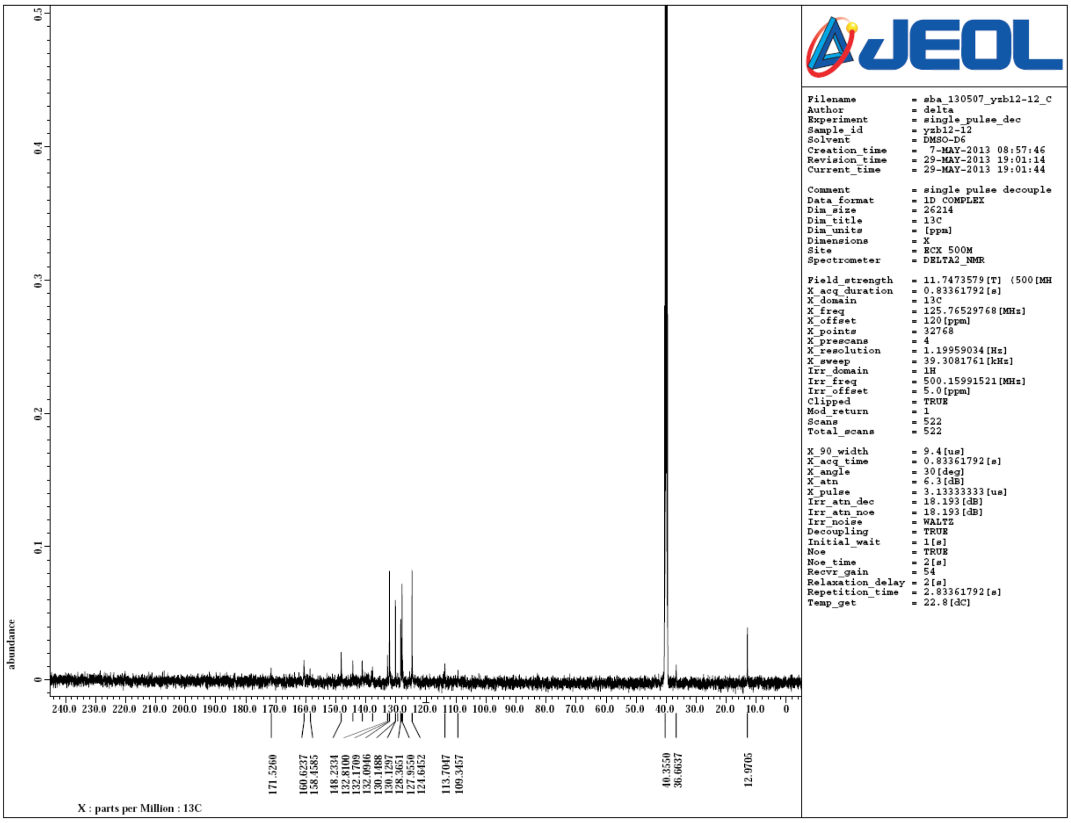

^{13}C NMR for compound **6i**

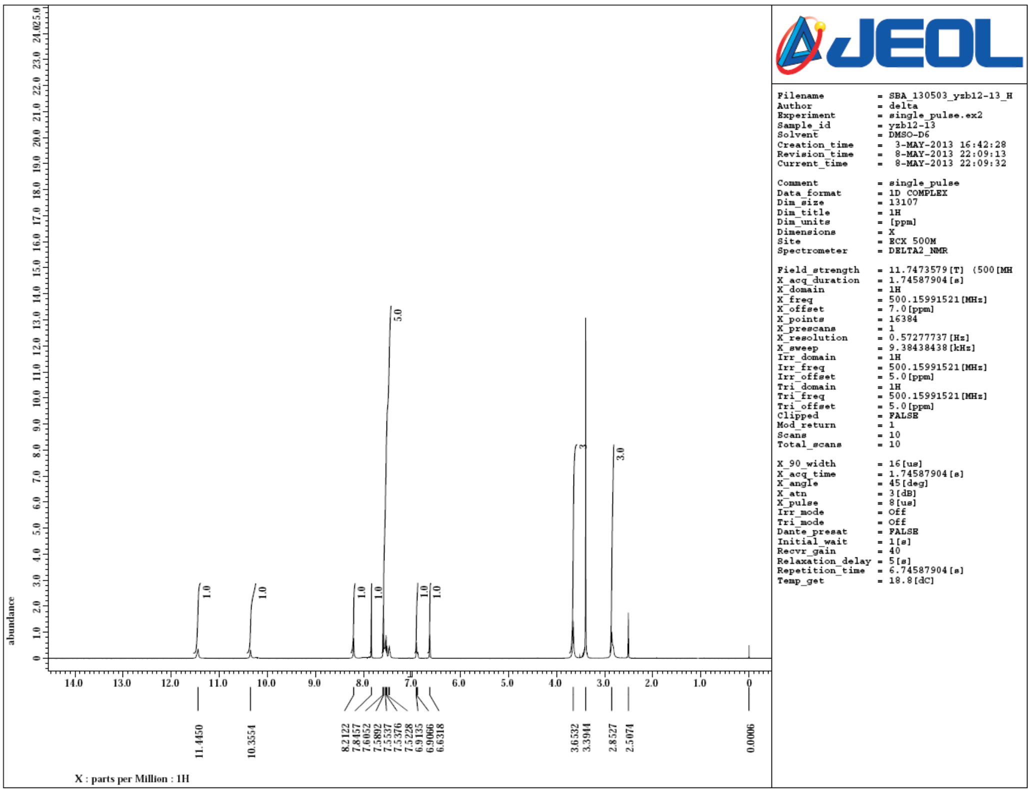

^{1}H NMR for compound **6j**

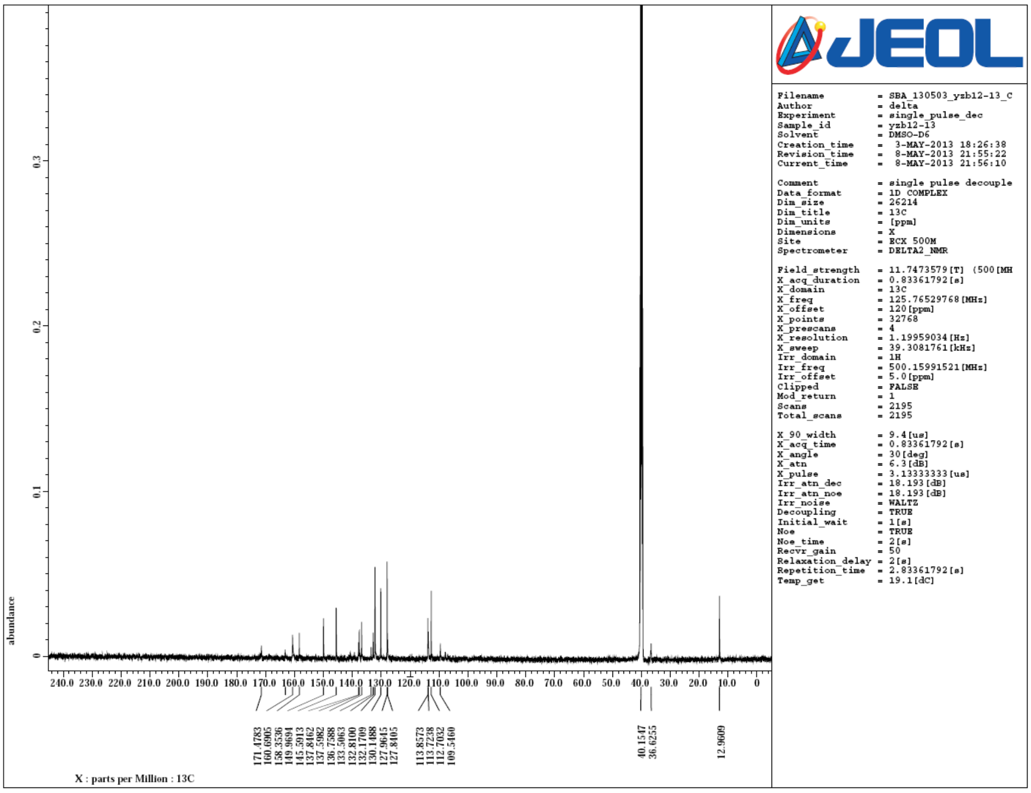

^{13}C NMR for compound **6j**

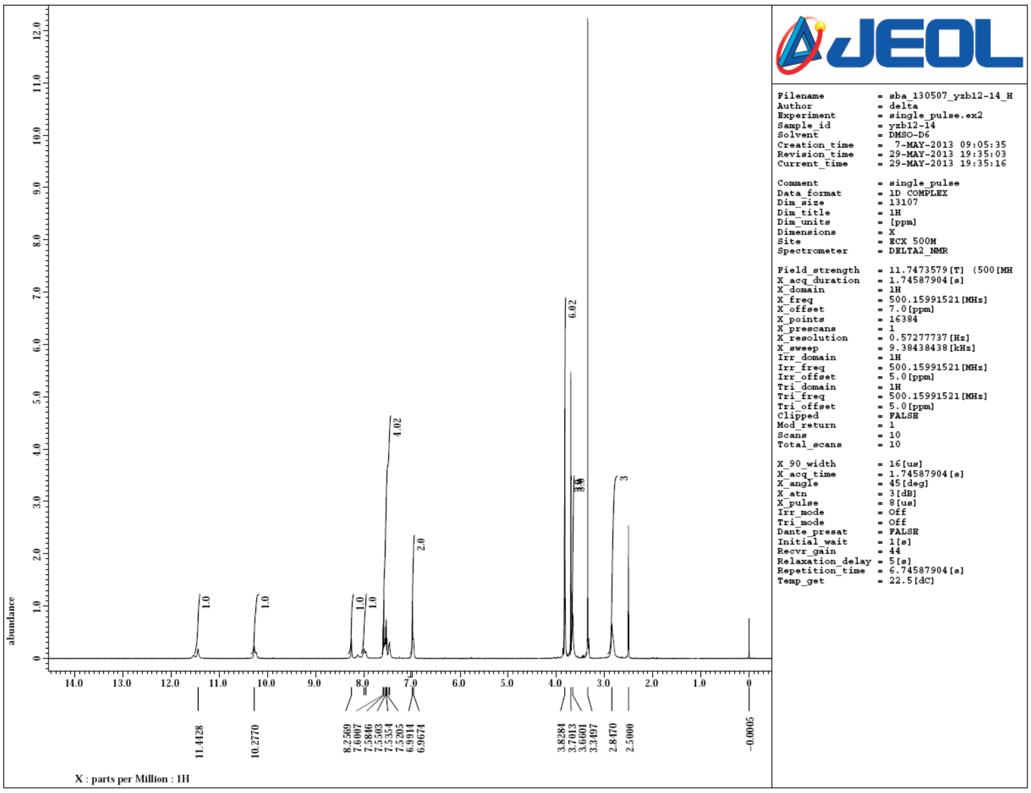

^{1}H NMR for compound **6k**

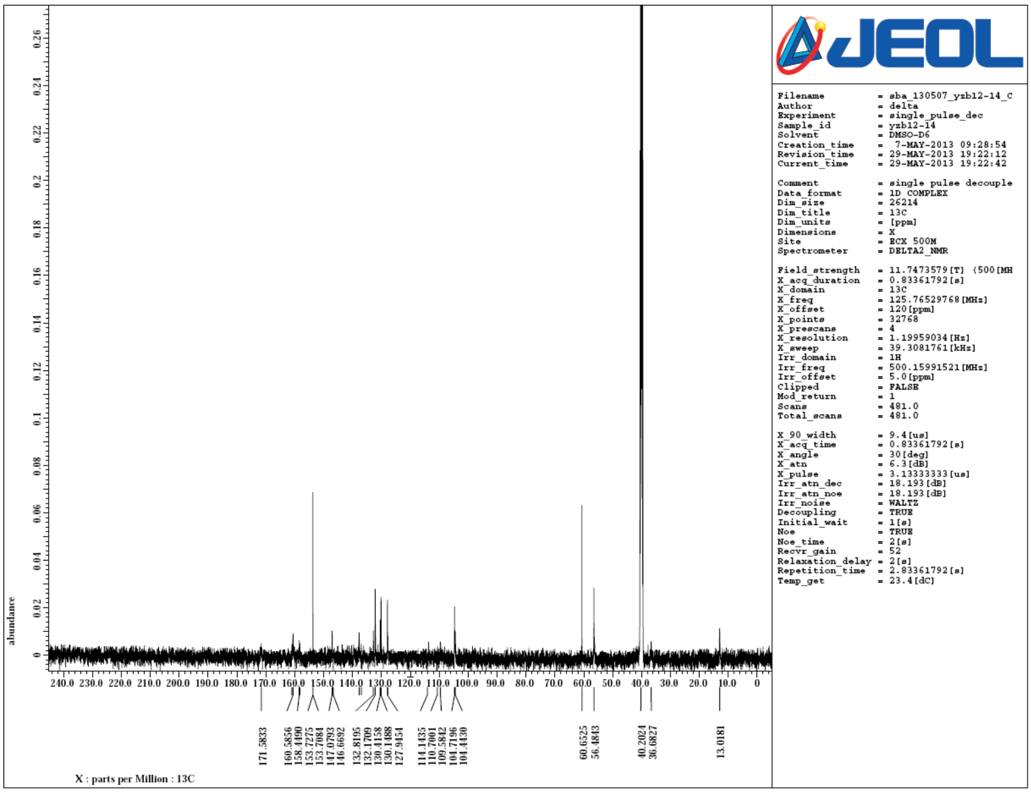

^{13}C NMR for compound **6k**

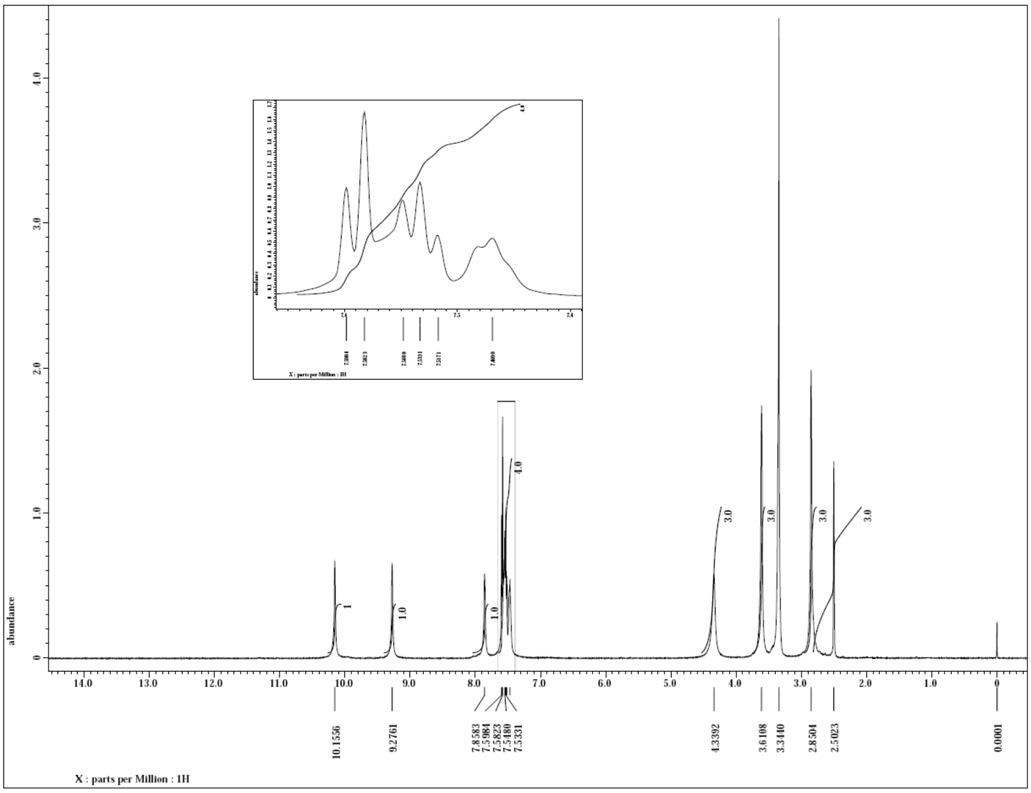

^{1}H NMR for compound **6l**

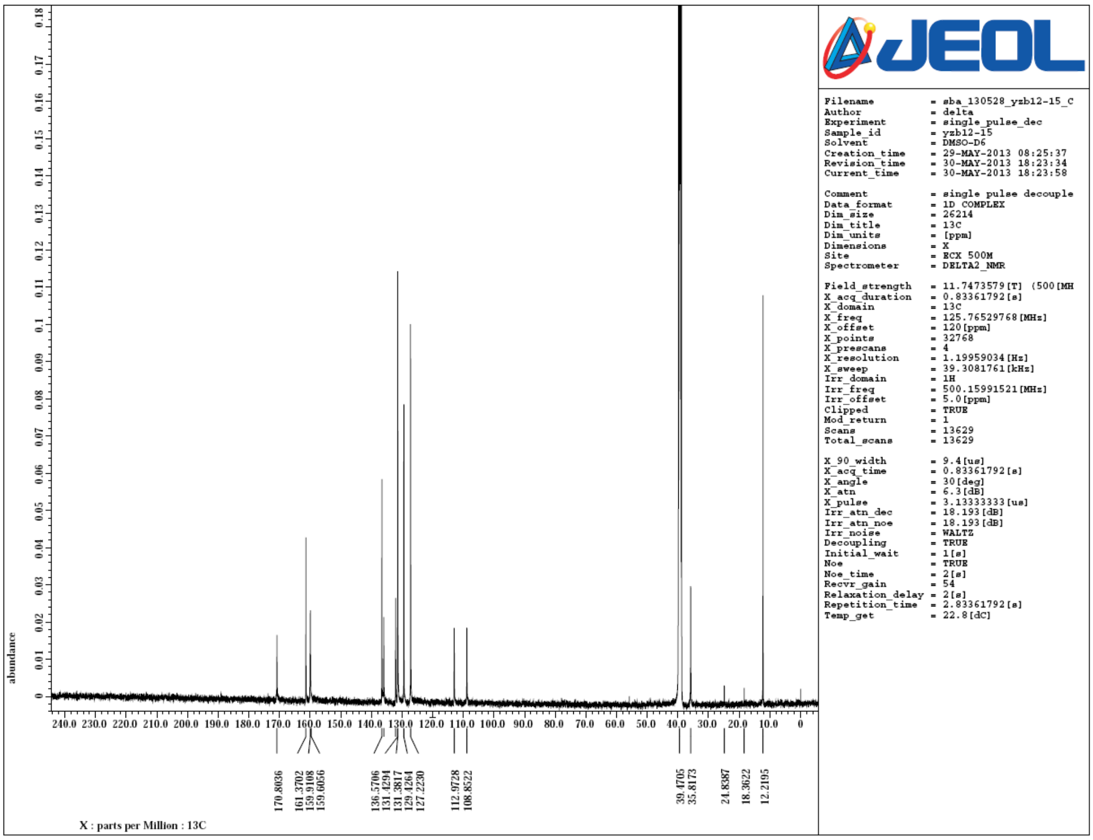

^{13}C NMR for compound **6l**

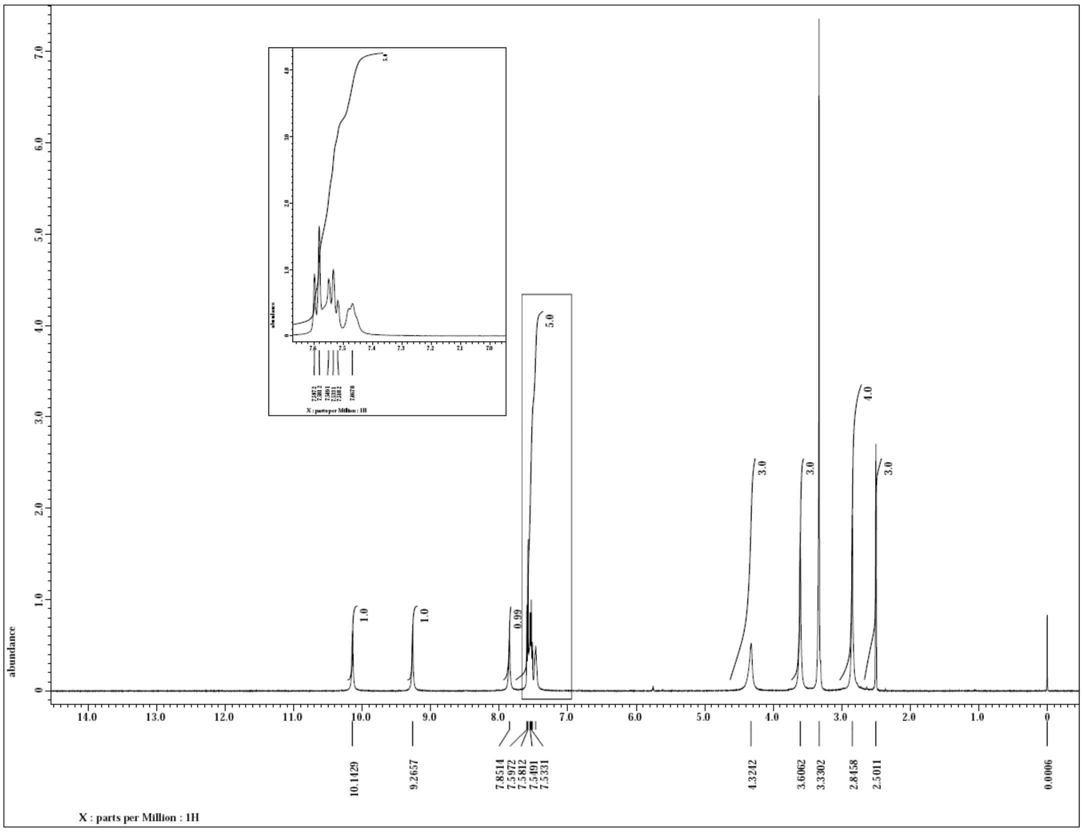

^{1}H NMR for compound **6m**

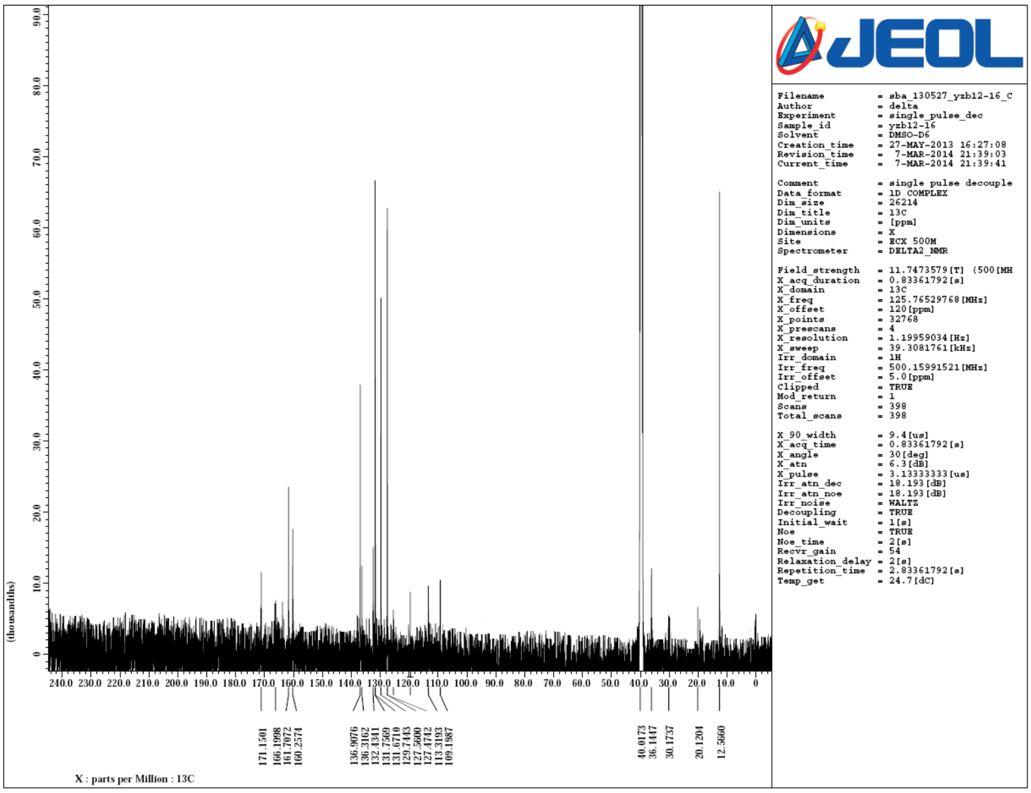

^{13}C NMR for compound **6m**

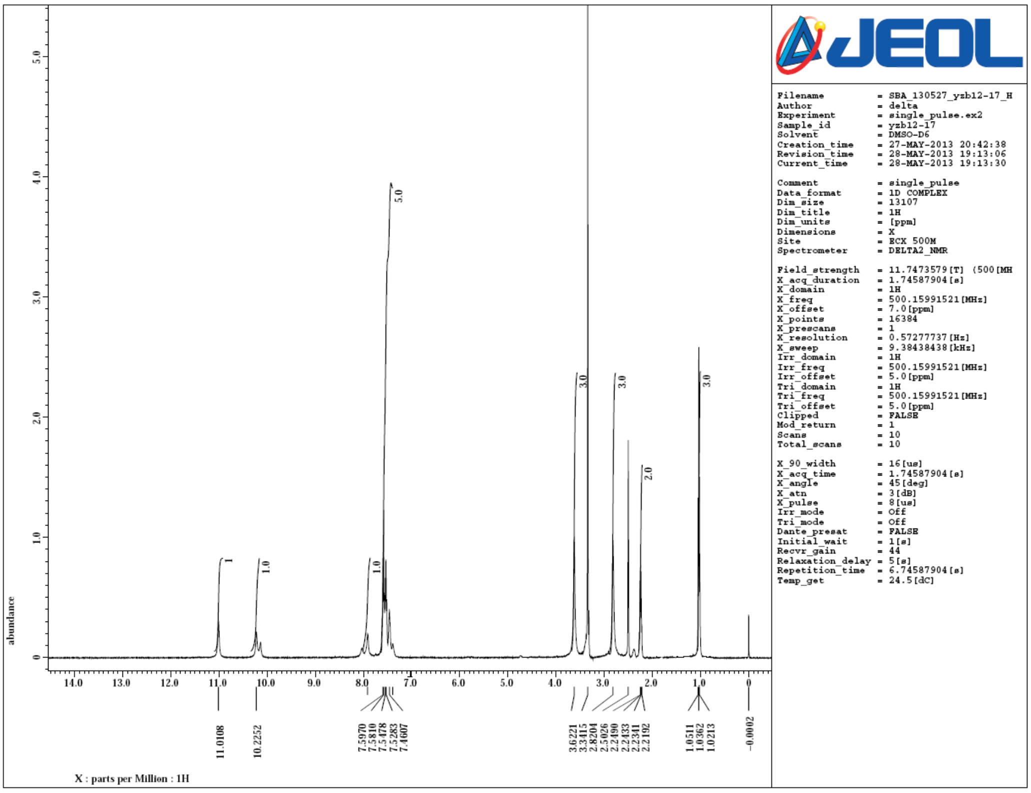

^{1}H NMR for compound **6n**

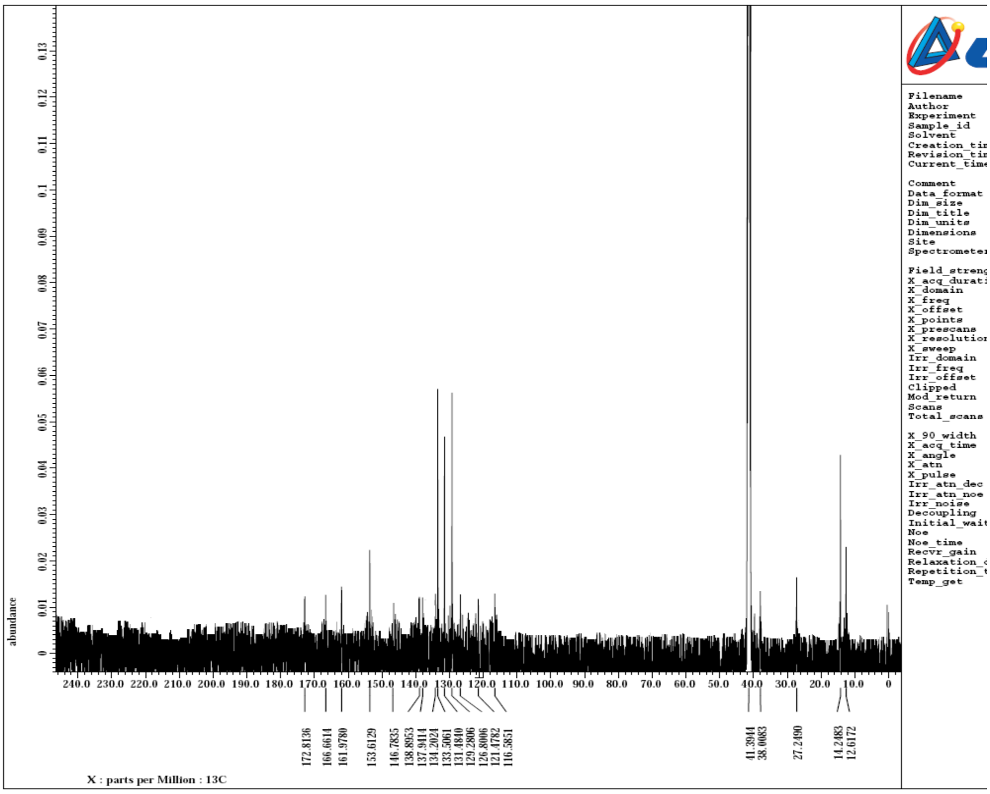

^{13}C NMR for compound **6n**

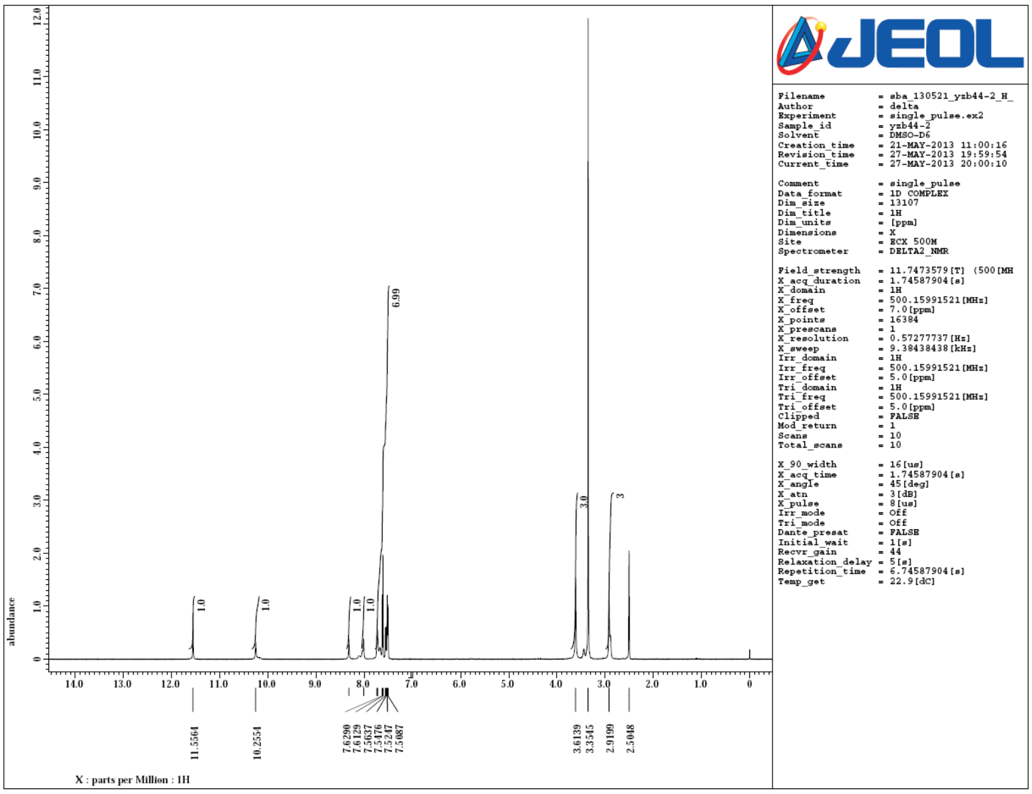

^{1}H NMR for compound **6o**

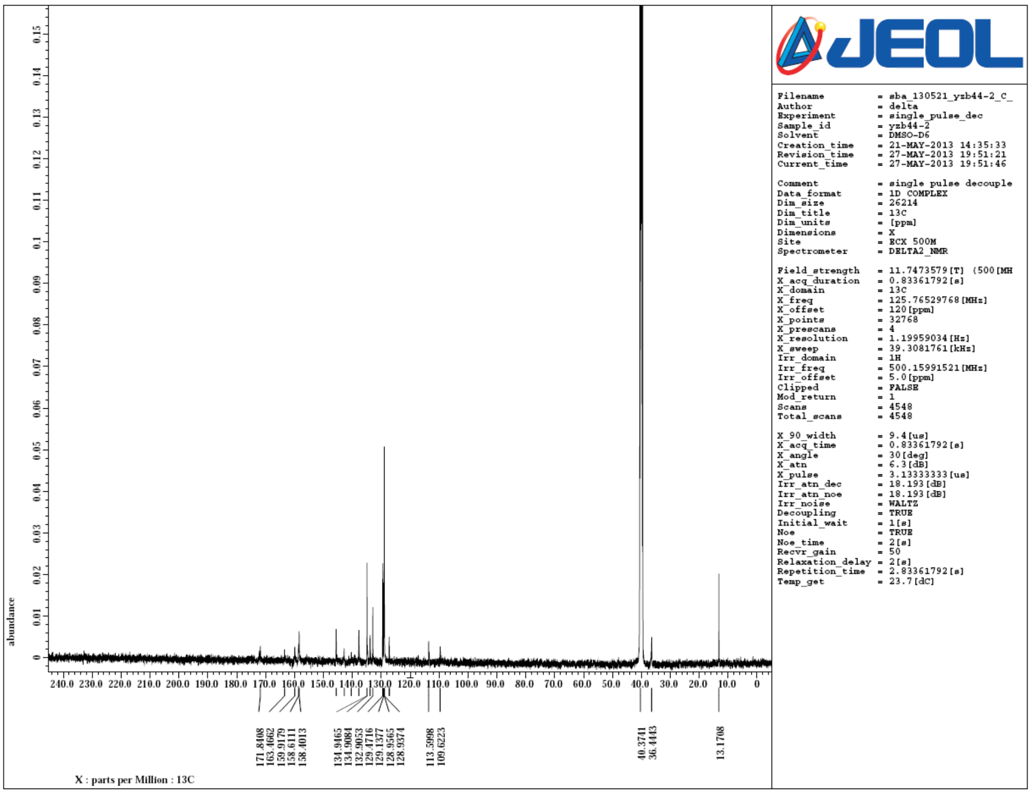

^{13}C NMR for compound **6o**

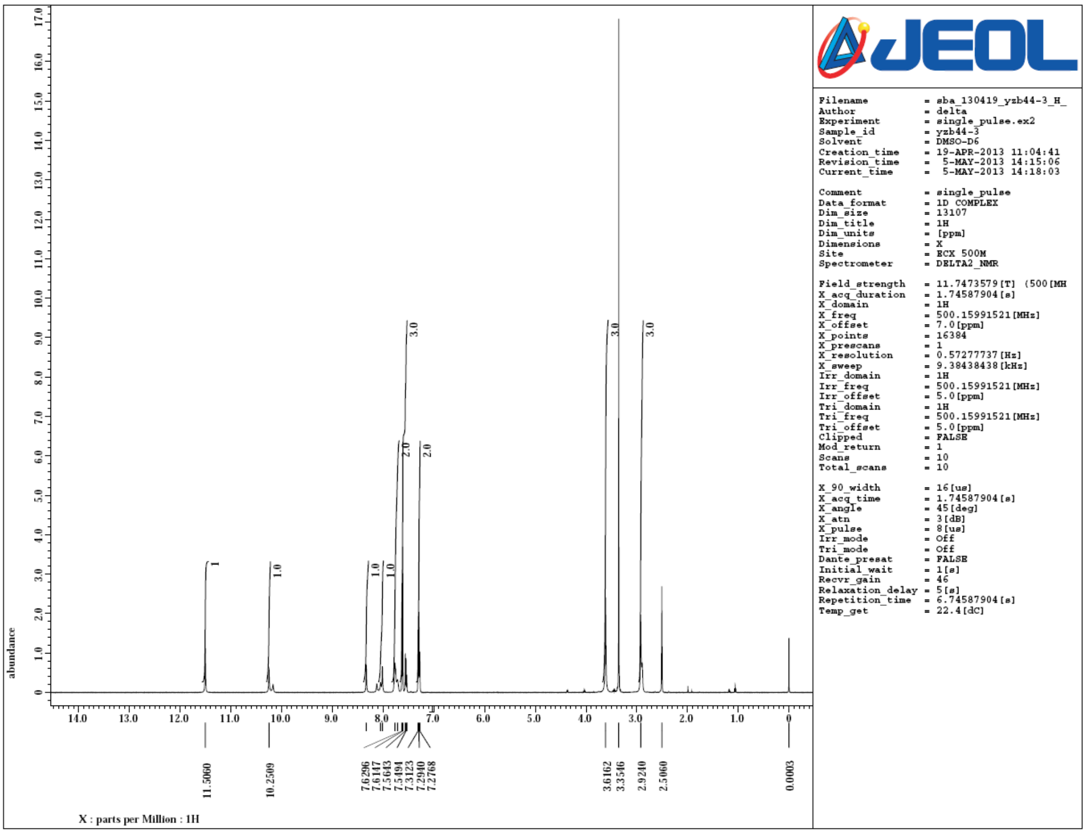

^{1}H NMR for compound **6p**

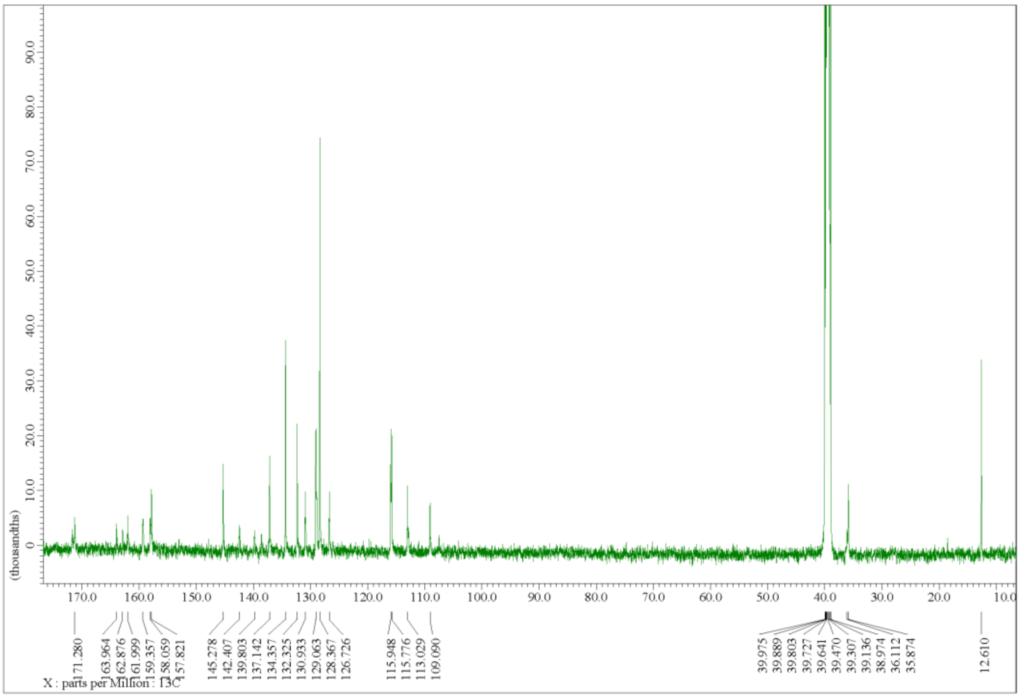

^{13}C NMR for compound **6p**

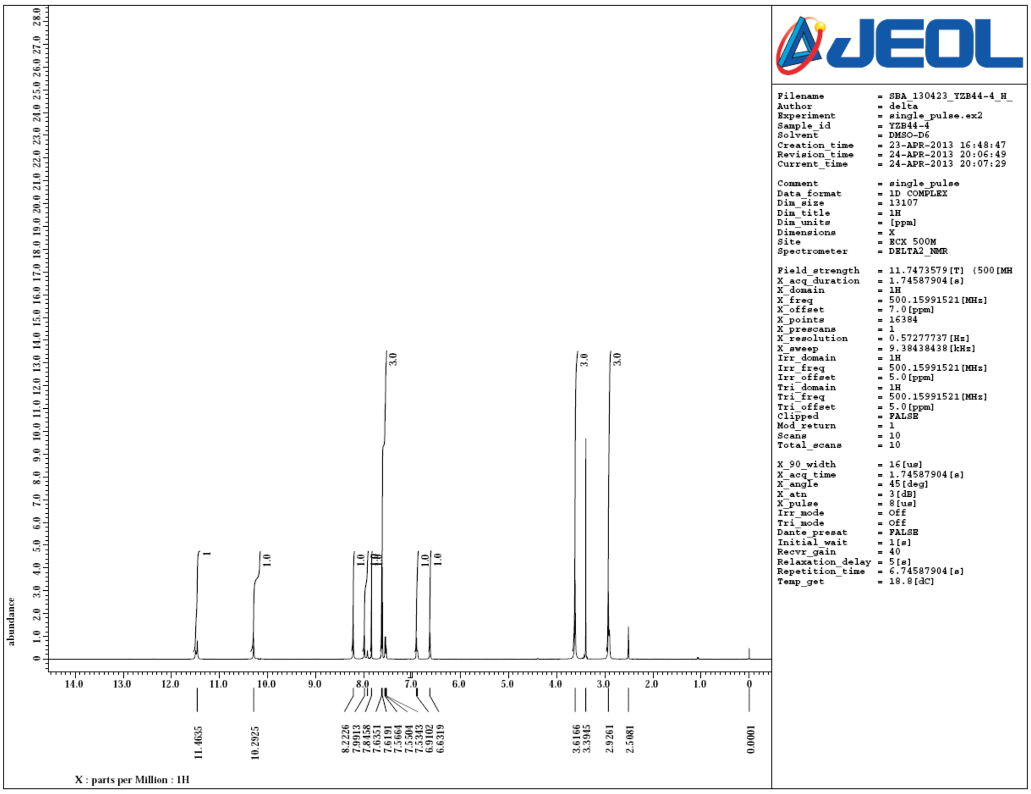

^{1}H NMR for compound **6q**

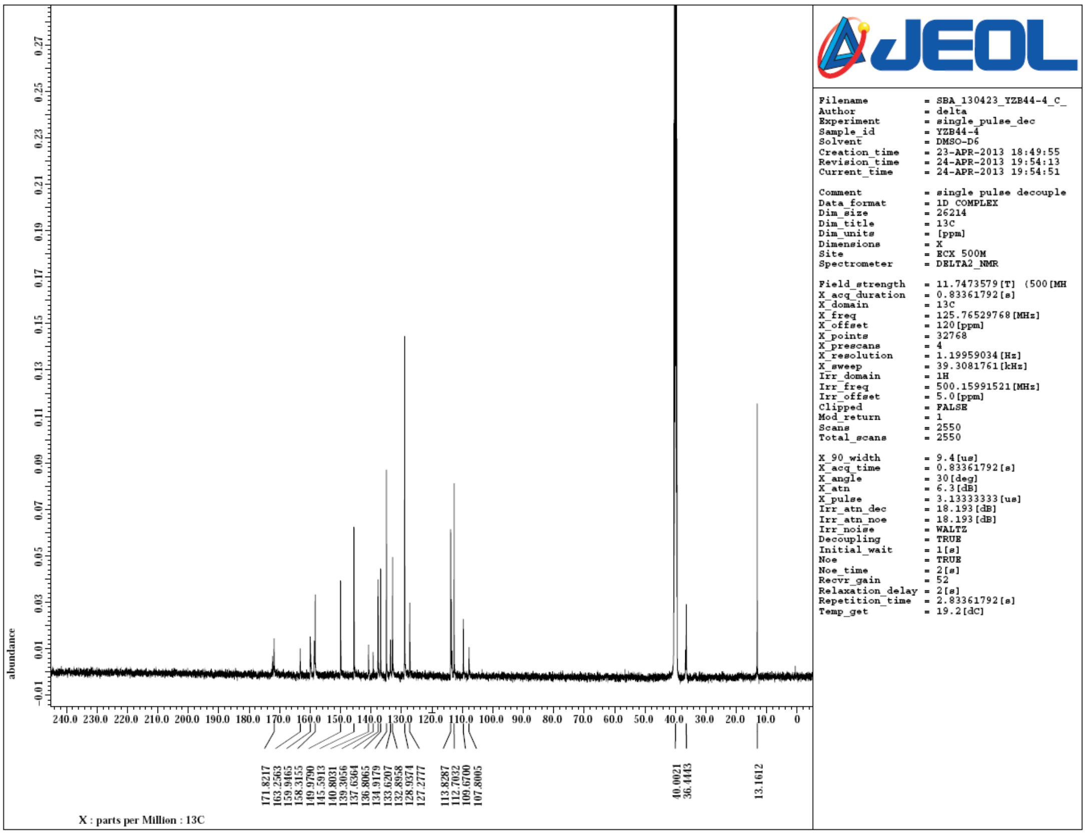

^{13}C NMR for compound **6q**

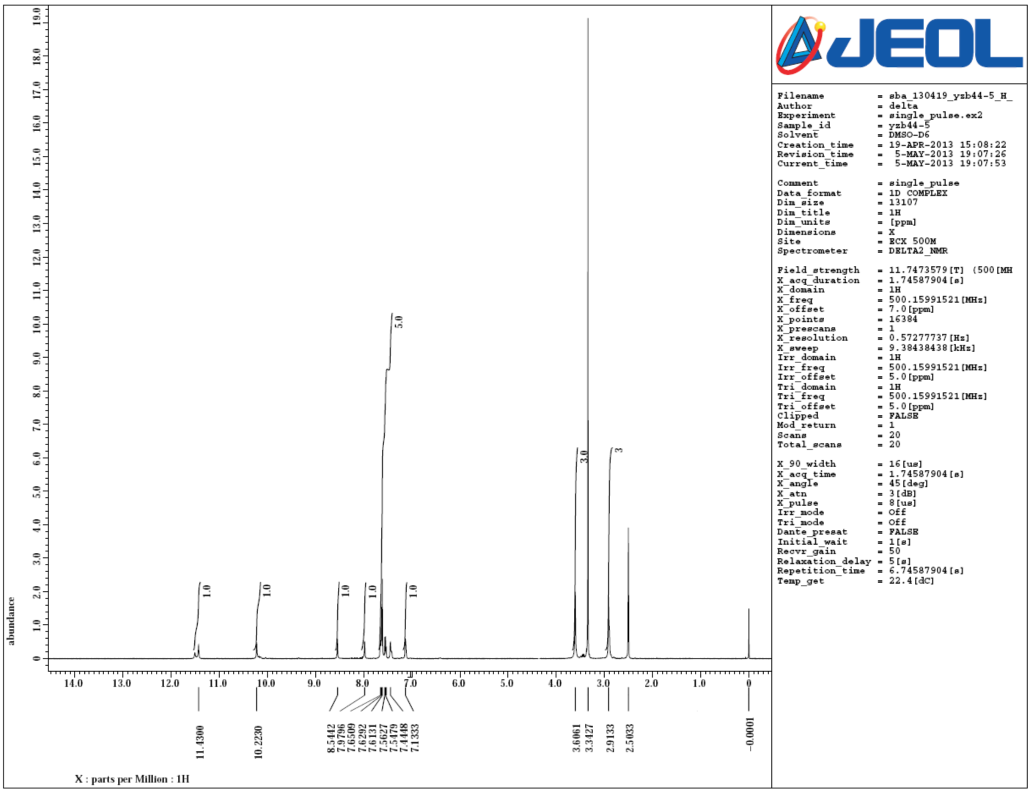

^{1}H NMR for compound **6r**

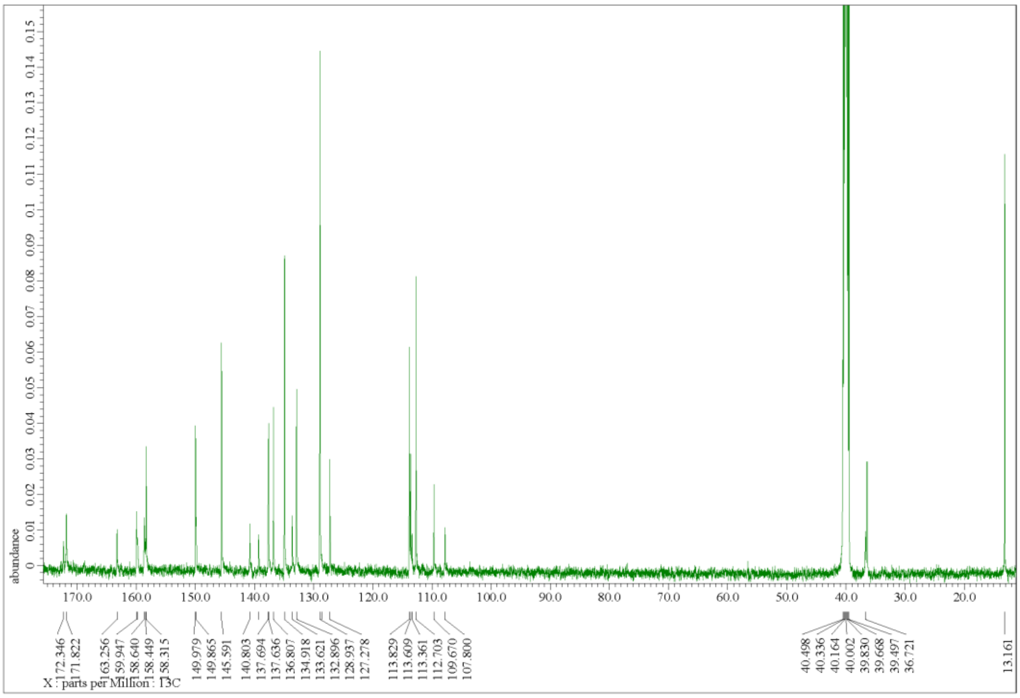

^{13}C NMR for compound **6r**
